# *StrokeClassifier:* Ischemic Stroke Etiology Classification by Ensemble Consensus Modeling Using Electronic Health Records

**DOI:** 10.21203/rs.3.rs-3367169/v1

**Published:** 2023-10-31

**Authors:** Ho-Joon Lee, Lee H. Schwamm, Lauren Sansing, Hooman Kamel, Adam de Havenon, Ashby C. Turner, Kevin N. Sheth, Smita Krishnaswamy, Cynthia Brandt, Hongyu Zhao, Harlan Krumholz, Richa Sharma

**Affiliations:** 1 Department of Genetics and Yale Center for Genome Analysis, Yale School of Medicine, New Haven, CT; 2 Department of Neurology and Comprehensive Stroke Center, Massachusetts General Hospital and Harvard Medical School Boston, MA; 3 Department of Neurology, Yale School of Medicine, New Haven, CT; 4 Department of Neurology, Weill Cornell Medicine, New York City, NY; 5 Departments of Genetics and Computer Science, Yale School of Medicine, New Haven, CT; 6 Department of Biomedical Informatics and Data Science, Yale School of Medicine, New Haven, CT; 7 Departments of Biostatistics, Yale School of Public Health, New Haven, CT; 8 Department of Internal Medicine, Yale School of Medicine, New Haven, CT

## Abstract

Determining the etiology of an acute ischemic stroke (AIS) is fundamental to secondary stroke prevention efforts but can be diagnostically challenging. We trained and validated an automated classification machine intelligence tool, *StrokeClassifier*, using electronic health record (EHR) text data from 2,039 non-cryptogenic AIS patients at 2 academic hospitals to predict the 4-level outcome of stroke etiology determined by agreement of at least 2 board-certified vascular neurologists’ review of the stroke hospitalization EHR. *StrokeClassifier* is an ensemble consensus meta-model of 9 machine learning classifiers applied to features extracted from discharge summary texts by natural language processing. *StrokeClassifier* was externally validated in 406 discharge summaries from the MIMIC-III dataset reviewed by a vascular neurologist to ascertain stroke etiology.

Compared with stroke etiologies adjudicated by vascular neurologists, *StrokeClassifier* achieved the mean cross-validated accuracy of 0.74 (±0.01) and weighted F1 of 0.74 (±0.01). In the MIMIC-III cohort, the accuracy and weighted F1 of *StrokeClassifier* were 0.70 and 0.71, respectively. SHapley Additive exPlanation analysis elucidated that the top 5 features contributing to stroke etiology prediction were atrial fibrillation, age, middle cerebral artery occlusion, internal carotid artery occlusion, and frontal stroke location. We then designed a certainty heuristic to deem a *StrokeClassifier* diagnosis as confidently non-cryptogenic by the degree of consensus among the 9 classifiers, and applied it to 788 cryptogenic patients. This reduced the percentage of the cryptogenic strokes from 25.2% to 7.2% of all ischemic strokes.

*StrokeClassifier* is a validated artificial intelligence tool that rivals the performance of vascular neurologists in classifying ischemic stroke etiology for individual patients. With further training, *StrokeClassifier* may have downstream applications including its use as a clinical decision support system.

## Introduction

Identifying the etiology of an ischemic stroke is a clinically challenging and consequential task. In the United States, there are nearly 676,000 cases of ischemic stroke per year^1^, a quarter of whom have had a prior stroke^2^. Among stroke survivors, another stroke can lead to death or further disability. The causative mechanism or etiology of an ischemic stroke can be heterogeneous including large artery atherosclerosis, cardioembolism, small vessel disease, and other rare, determined etiologies^3^. Nearly 20–30% of ischemic stroke patients in the U.S. are considered cryptogenic with no etiology determined after evaluation^4–11^. The risk of recurrent stroke after a cryptogenic stroke is heightened at 5.6% at 3 months and between 14–20% at 2 years^12,13^. In one study, at 21 months, cryptogenic strokes were associated with higher risk of recurrent stroke in comparison with cardioembolic (HR 1.83, p=0.028) and non-cardioembolic stroke patients with known source (HR 2.4, p=0.046). An analysis of the NOR-FIB study demonstrated an annual risk of stroke recurrence of 7.7% versus 2.8% among individuals with cryptogenic versus non-cryptogenic strokes, respectively^14^. In the Athens Stroke Registry, the stroke recurrence rate in patients with cryptogenic stroke was 29% over a mean of 30.5 months, significantly higher compared with all non-cardioembolic stroke subtypes^15^.

The diagnosis of ischemic stroke etiology determined by a patient’s treating clinician may partly contribute to the differential rates of stroke recurrence by etiology as each diagnosis prompts a specific secondary stroke prevention treatment plan. Evidence-based, etiology-specific treatments that are proven to reduce the risk of recurrent stroke to varying degrees include carotid revascularization for symptomatic severe carotid stenosis, anticoagulation for atrial fibrillation or left ventricular thrombus, dual antiplatelet therapy after intracranial stenosis-related stroke, and patent foramen ovale closure when it is implicated, among others^16^ (Supplementary Information). Despite high-level evidence supporting the efficacy of such therapies to prevent recurrent stroke, secondary stroke prevention treatments are significantly underutilized both in the U.S. and globally after an ischemic stroke^17–20^. This implementation gap may underlie the observation that the majority of recurrent strokes are from the same etiology as the index stroke^21^. Furthermore, a cryptogenic stroke diagnosis precludes the institution of any guideline-recommended therapy that targets specific stroke mechanisms and reduce the risk of recurrent stroke from culprit sources^16^. The ability to tailor and implement secondary stroke prevention strategies fundamentally hinges on the diagnosis of the culprit mechanism of an ischemic stroke.

To determine the causative mechanism of an ischemic stroke, clinicians synthesize a vast array of data including clinical history and physical examination, laboratory data, cardiac rhythm interrogation, cardiac imaging, and neuroradiologic studies. Utilization of diagnostic tools has increased with time, nevertheless, a significant proportion of patients remain cryptogenic^22^. Diagnostic uncertainty arises due to 1) an inadequate or incomplete workup with further results pending after discharge, 2) a complete workup yielding no known stroke etiology, or 3) multiple, competing possible etiologies, resulting in a diagnosis of stroke of undetermined etiology^3^. An exacerbating factor may be the lack of widespread neurovascular experts specifically trained to collect and examine data to ascertain stroke etiology. A study has demonstrated that compared to evaluation by a non-vascular neurologist, evaluation by a vascular neurologist was associated with a more comprehensive diagnostic investigation that may change management^23^. There is a shortage of vascular neurologists in the United States, with only one in every 6 ischemic stroke patients treated by a board-certified vascular neurologist^23^. In this context, there is an opportunity for an automated, artificial intelligence solution to standardize the process of diagnosing the causative mechanism of stroke.

Artificial intelligence has been heavily adapted for clinical use to help determine patient eligibility for acute stroke therapies such as thrombectomy to abort a stroke, but only minimally for the purpose of stroke prevention^24–26^. There have been several studies of machine learning classifiers to predict stroke etiology, however these have been limited by use of manually curated discrete features, single center samples, insufficient adjudication of stroke etiology outcomes, exclusion of patients with multiple potential etiologies, reliance on a singular model, lack of model explainability, or broad, heterogeneous categorization of stroke etiology^27–33^. In this multi-center study, we aim to develop and externally validate a multi-level, automated ischemic stroke etiology classifier by applying natural language and innovative machine learning tools applied directly to semi-structured text data from the EHR compiled during the AIS hospitalization.

## Methods

### Study Population and Data Sources

The derivation cohort consisted of hospitalizations at 2 academic, Comprehensive Stroke Centers of Yale New Haven Hospital (YNHH) and Massachusetts General Hospital (MGH) from 2015 to 2020. Institutional Review Board approval was obtained from both YNHH and MGH. The external validation cohort was a subgroup of hospitalizations at the academic, Comprehensive Stroke Center of Beth Israel Deaconess Medical Center from 2001 to 2012. Access to this cohort’s data was obtained through the MIMIC-III (Medical Information Mart for Intensive Care) warehouse which contains records of 46,520 hospitalizations from 2001 to 2012 at Beth Israel Deaconess Medical Center. MIMIC-III is a publicly available, de-identified health record repository that was developed and approved by the Beth Israel Deaconess Medical Center and Massachusetts Institute of Technology IRBs^34^. Two of the authors (H.L. and R.S.) were approved to have access to this database for research after passing the requisite training course^34,35^.

Acute ischemic stroke hospitalizations at YNHH and MGH were identified by each institution’s Get-with-the-guidelines stroke database. Get-With-The-Guidelines (GWTG)-Stroke database is a quality improvement initiative in which participating hospitals enter clinical and radiographic data of all patients hospitalized with an ischemic stroke diagnosis^36^. Acute ischemic stroke patients are identified by administrative billing codes (International Classification of Diseases (ICD), 10th Revision). Data abstraction, entry, and adjudication are performed by trained study personnel. There are logic checks and form controls to minimize data entry errors. The database was queried for all ischemic stroke patients ≥18 years admitted from January 2015 to December 2020 at MGH and YNHH to assemble the ischemic stroke cohort. The EHR platform for both institutions is Epic (Epic Systems Corporation), the most prevalent EHR system in the United States. Stroke hospitalizations from the GWTG databases were linked with corresponding semi-structured discharge summary plain ASCII text files, resulting in a total 1,269 and 1,493 records from YNHH and MGH, respectively.

The MIMIC-III dataset was queried for the ICD-9 codes of 433.X and 434.X that are associated with ischemic stroke, resulting in a total of 2,563 hospitalization records from patients ages >18 years admitted to BIDMC from 2001 to 2012. A subset of these, a convenience sample of the first consecutive 500 records, were included in this study for external validation and their discharge summary plain ASCII text files were analyzed. BIDMC utilizes its own customized, hospital-wide EHR system. A description of the study populations from the 3 institutions represented in this analysis is provided in Table 1.

### Outcomes

The primary study outcome was stroke etiology as defined by the 5 mutually exclusive causative mechanisms of stroke per the TOAST classification system: 1- large artery atherosclerosis, 2- cardioembolism, 3- small vessel disease, 4- other determined etiology, and 5- undetermined etiology (cryptogenic)^3^. Stroke etiology was determined by the agreement of two board-certified vascular neurologists. The first vascular neurologist was the discharging treating clinician, when applicable, who documented a stroke etiology impression in the EHR. The second vascular neurologist was the study co-author (R.S.) who reviewed the entire stroke hospitalization record and viewed the neuroimaging. When either there was disagreement about the stroke etiology between the two vascular neurologists or the discharging treating clinician was not a vascular neurologist (4% and 2% of the YNHH and MGH cohort, respectively), a third vascular neurologist at each of the two institutions (A.D. and A.C.T. at YNHH and MGH, respectively) reviewed the entire stroke hospitalization record and provided stroke etiology diagnosis impressions. The final stroke etiology diagnosis was the etiology ascribed by the majority. If there was no majority, the stroke etiology diagnosed by the senior-most vascular neurologist was utilized. In the external validation cohort, the co-author, R.S., reviewed the text of each discharge summary and designated a TOAST classification based on the data recorded in the text corpus.

### Covariates

#### Demographic Variables-

Using regular expressions, we extracted age and sex from discharge summary text. The YNHH dataset did not contain sex information in a structured format in the discharge summary, unlike the MGH data. To identify sex information from the YNHH data, we used a customized R code to search for “her” or “his” in the EHR texts to assign female or male to each EHR, respectively. We compared the accuracy of this extraction with the age and sex fields hardcoded in the corresponding institutional GWTG-stroke registry. We intentionally did not include the proxy variable of race as a covariate for model training and testing because our datasets lack measures of the social environment which may be more relevant indicators of stroke etiology than ancestry alone^37^.

#### Clinical Variables Derived from MetaMap-

We applied natural language processing tools to the corpus of discharge summary texts to engineer clinical variables that may associate with stroke etiology. Firstly, discharge summaries were processed by the natural language processing (NLP) or text mining tool, *MetaMap*, developed by the National Library of Medicine (NLM) to extract terms from text and link them to standard biomedical concepts in the Unified Medical Language System (UMLS) Metathesaurus^38,39^. Each discharge summary is a semi-structured text that can be processed by MetaMap to detect unique concepts or concept unique identifiers (CUIs) from the UMLS which contains over 1 million biomedical concepts in an automated manner. We applied MetaMap to the discharge summary text of each hospitalization and extracted CUIs that belong to the following 3 types or categories: “Disease or Syndrome”, “Neoplastic Process”, and “Sign or Symptom” (**Table S1**). The rationale for selecting MetaMap CUIs was that it was designed to retrieve medical concepts by lexical analysis and tokenization. MetaMap allows for abbreviations, acronyms, negations, and parts-of-speech tagging. It facilitates lookups in the SPECIALIST system that is supported by the UMLS Metathesaurus and Semantic Network, a repository of biomedical concepts and their interrelationships^40^ that is updated quarterly and incorporates SNOMED CT content which is routinely utilized in SNOMED CT-enabled EHR systems to enable meaning-based retrieval of information and maps to ICD-9 and ICD-10 coding systems^41^. MetaMap also performs word sense disambiguation by which concepts are favored if semantically consistent with surrounding text. There is also flexibility in input and output data formats permissible by MetaMap. Finally, MetaMap has been rigorously tested in various biomedical research applications^42,43^. Compared with other clinical entity extraction tools, MetaMap was demonstrated to have the highest recall and F1-score when tasked with identifying clinical concepts such as obesity-related symptoms^44^. In one study, MetaMap extracted biomarker types from pathology reports with > 95% accuracy^45^. To our knowledge, this is the first application of MetaMap in stroke research.

### Other Variables:

By employing customized regular expressions, we curated 4 other categories of features from discharge summaries (**Table S2**). First, we extracted clinical information not captured by CUIs including social history (tobacco, ethanol, and illicit drug use), National Institutes of Health Stroke Severity scale, and vital signs, which we designate as 6 HEX features. Second, we extracted 40 radiologic features (RAD) from studies performed during the stroke hospitalization including information about the neuroanatomical location of the ischemic stroke, the presence of moderate or severe stenosis or occlusion of specific head and neck arteries, and the occurrence of intracranial hemorrhage encoded as a binary variable. The accuracy of our automated method of radiology data extraction in a random sample of 100 selected for each variable was 98% for neuroanatomic location and 99% for vessel abnormality^46^. Third, we also extracted 36 cardiac features (HRT) from electrocardiography and echocardiography reports in the discharge summary. Finally, we extracted 18 laboratory features (LAB). In a random sample of 5 YNHH and 5 MGH patients, the accuracy of the HRT and LAB features that were extracted was 100%. In order to reduce measurement noise or error, the following cutoff values were used to discretize values of the HEX and LAB features for 2 levels with 1 cutoff (high and normal) or 3 levels with 2 cutoffs (high, normal, and low): (1) Ejection fraction, 40 (2) NIHSS, 6 (3) Sodium, 136 (4) BUN, 24 (5) ALT, 36 (6) AST, 36 (7) White blood cell count, 11 (8) Hematocrit, 46 and 35 (9) Hemoglobin for female, 15.5 and 11.7 (10) Hemoglobin for male, 17.1 and 13.2 (11) Triglycerides, 200 (12) HDL, 40 (13) LDL, 100 (14) TSH, 4.2 (15) PTT, 29.9 (16) A1C, 6.5. We denote the discretized feature groups by HEXd and LABd. We assess model performance based on each of the 5 feature groups, all the 5 groups, or those 5 combinations excluding each group. We assess completeness of the investigation for stroke etiology during the hospitalization based on values available for each of these groups.

### Imputation of missing data

We deployed a multiple imputation method, MICE (Multivariate Imputation by Chained Equations)^47,48^, from the *mice* package in R to impute missing values in categorical and numerical features of the YNHH and MGH data using the built-in method of predictive mean matching (*pmm*) with the default parameters. We also imputed the missing MIMIC features using the built-in method of Random Forests (*rf*; with the default parameters), which we found was better to deal with larger fractions of missing values than *pmm* or other built-in imputation methods.

### Dimensionality reduction of features by principal component analysis

Since the number of features totaled 2,027, we explored the relationship between dimensionality reduction of features and model training and performance. We chose principal component analysis (PCA) to reduce the feature dimensionality because of its clear interpretation of each principal component as a linear combination of all features. We applied PCA to all features and selected the top PCs for each of the following 10 thresholds of the total variance: 10%, 20%, 30%, 40%, 50%, 60%, 70%, 80%, 90%, 95%, and 99%. Validation and test datasets were transformed based on PCA of training datasets.

### Machine learning model development and evaluation

**Figure 1.**
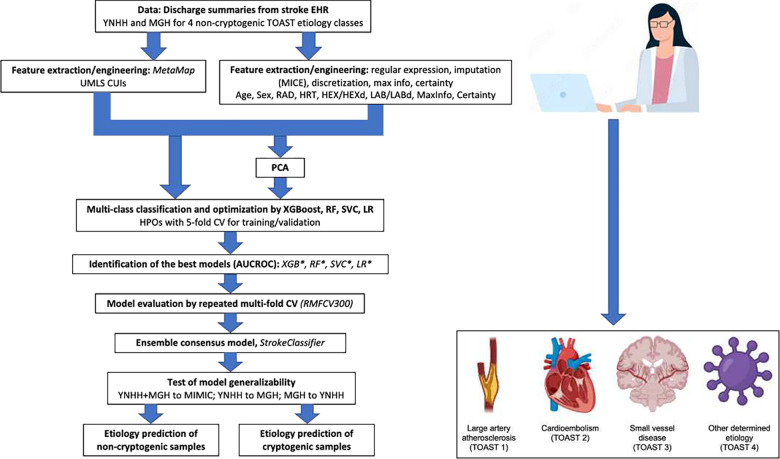
Workflow overview. Icons were created with BioRender.com.

We analyzed non-cryptogenic ischemic stroke hospitalization records of discharge summaries from the merged YNHH and MGH datasets for model training and validation. Figure 1 shows an overview of our workflow. Records from non-cryptogenic ischemic stroke hospitalizations in the MIMIC dataset were used as the test dataset. We built models using the following 20 different feature groups individually: CUIs; RAD; HRT; HEX; HEXd; LAB; LABd; RAD + HRT + HEX + LAB, CUIs + HRT + HEX + LAB, CUIs + RAD + HEX + LAB, CUIs + RAD + HRT + LAB, CUIs + RAD + HRT + HEX, CUIs + RAD + HRT + HEXd, CUIs + RAD + HRT + HEX + LAB, and CUIs + RAD + HRT + HEXd + LABd. For the last two groups, we also applied a filtering of samples based on maximum information (MaxInfo) ≥ 4 (the number of feature categories present) and the 11 PCA-based feature groups described above.

We built base models using 4 different supervised machine learning algorithms to classify the 4-level non-cryptogenic stroke etiology outcome: logistic regression (LR), support vector classifier (SVC), Random Forests (RF), and XGBoost (XGB). Each model was optimized with a grid search of a pre-defined hyperparameter space for each of 24 training datasets, i.e., a total of 96 (= 4*24) hyperparameter optimization (HPO) runs, and a stratified cross-validation (CV) strategy of 5 splits of 20% validation sets using *StratifiedShuffleSplit* from the *scikit-learn* library in Python. We controlled the randomness of the stratified CV by setting the parameter, *random_state* = 1701, in this work. The best models with optimized parameters were selected based on the maximum AUCROC (the area under the curve of the receiver operating characteristic). Details about models and configurations for HPO are provided in Supplementary Information. For the 4 best models with the optimal parameters identified by the above strategy, we next performed more comprehensive training and validation using a repeated multi-fold CV strategy to minimize statistical bias and ensure robustness compared to the single 5-fold CV strategy above. We performed 2-fold, 3-fold, 4-fold, 5-fold, and 10-fold CV with 30, 20, 15, 12, and 6 repetitions with different random seeds, respectively (using *RepeatedStratifiedKFold* from the *scikit-learn* library in Python), i.e., 60 * 5 = 300 CV experiments in total. We denote this strategy as *RMFCV300*.

Next, we built 4 ensemble models using the 4 optimized models selected above as well as SVC with alternative prediction probabilities, which we term *SVC2* (Supplementary Information), as base models. The rationale for building ensemble models is that ensemble learning has demonstrated success in improving performances over single models in reducing variance or bias^49–51^. From predicted probabilities generated from these 5 base models, the mean, median, maximum, and minimum for each class were normalized across the 4 classes as 4 ensemble models: MEAN, MEDIAN, MAX, and MIN, respectively. Our summary-statistic-based ensemble models are a variant of stacked generalization^52^ without additional training. This yielded a 9-classifier system of 5 optimized base and 4 ensemble classifiers. We obtained consensus predictions among those 9 classifiers as a meta-classifier or a voting system to reduce or average out any bias from a single classifier and improve robustness. The resulting algorithm was designated as *StrokeClassifier*. We additionally analyzed *StrokeClassifier* by (1) training on the YNHH dataset and testing on the MGH and MIMIC datasets and (2) training on the MGH dataset and testing on the YNHH and MIMIC datasets for a 5-way cross-hospital validation in total. For the purpose of comparison, we also employed stacking ensemble models using each of LR, SVC, RF, and XGB as a meta model (Supplementary Information). For model performance evaluation, we used the following 7 performance metrics based on weighted averages for one-vs-rest classification: AUCROC, area under the precision-recall curve (AUPRC or average precision), accuracy (i.e., weighted recall), balanced accuracy (i.e., macro recall or the arithmetic mean of sensitivity and specificity), precision, F1, and Cohen’s kappa. As for the qualitative interpretation of Cohen’s kappa values, we follow the scheme by Landis and Koch^53^: kappa < 0 as no agreement, 0 – 0.20 as slight, 0.21 – 0.40 as fair, 0.41 – 0.60 as moderate, 0.61 – 0.80 as substantial, and 0.81 – 1 as almost perfect agreement.

For model interpretation and feature importance, we performed the game-theoretic Shapley value-based SHAP (SHapley Additive exPlanations) analysis using the *shap* package in Python^54,55^, as in our previous works^56,57^. We used TreeSHAP for RF and XGB and KernelSHAP for LR and SVC with a k-means background with k = 100 for computational efficiency. As an alternative approach to ascertain feature importance, we performed classifier-agnostic Student’s t-tests and Kolmogorov-Smirnov tests for one-vs-rest comparisons for each class and each feature.

We performed exploratory analyses to evaluate etiologic predictions by *StrokeClassifier* for cryptogenic strokes adjudicated by vascular neurologists. We examined various certainty heuristics defined computationally by thresholds of diagnostic confidence. These diagnostic confidence thresholds were designated by the number of consensus supports provided by the 9 individual classifiers in the ensemble model for each non-cryptogenic stroke etiology. As a proof of concept, we applied the threshold of the first quartile of frequencies of support for each etiology from the external validation of the MIMIC-III cohort to predict the etiologies of cryptogenic patients (788 in total) and evaluated the distribution of predicted etiologies. Those predictions with the consensus frequencies less than the thresholds were deemed persistently cryptogenic. We also examined etiology distributions yielded by other quartile thresholds and the means of the support frequencies. Finally, using the first quartile thresholds, we identified a repertoire of EHR signatures associated with each predicted TOAST class for cryptogenic strokes by evaluating feature frequencies from *StrokeClassifier*.

All analyses were performed in Python and R using a macOS laptop with 2.6 GHz 6-Core Intel Core i7 and 32GB memory in the case of RF and LR and a high-performance computing cluster with 64 cores and 1GB memory per core in the case of XGB and SVC.

## Results

### Study Participants

The study sample consisted of 3,262 discharge summaries with AIS diagnoses (N=1,269 at YNHH from 2015–2020; N=1,493 at MGH from 2016–2019; N=500 at BIDMC from 2001–2012). The characteristics of the 3 cohorts are presented in Table 1. The derivation cohorts of YNHH and MGH were similar with some exceptions. The YNHH cohort was significantly older (median age 71 years [IQR 59–82]) compared with the MGH cohort (median age 69 [IQR 59–79]) (p=0.013). The median word count of the YNHH discharge summaries (1639 words [IQR 1274–2064]) was significantly lower than in the MGH discharge summaries (2058 words [IQR 1593–2554]) (p=1.21e-35). The YNHH cohort was significantly more likely than the MGH cohort to have hyperlipidemia (32.9% versus 11.5%, p=0.001) and coronary artery disease (17.8% versus 4.0%, p=0.003). The YNHH and MGH cohorts had similar distributions of stroke etiologies adjudicated by vascular neurologists: large artery atherosclerosis (19.8% versus 21.0%), cardioembolism (32.9% versus 29.9%), small vessel disease (15.3% versus 10.7%), other determined etiology (8.9% versus 9.6%), and cryptogenic etiology (23.1% versus 28.8%). The degree of completeness of extracted features was comparable between YNHH and MGH with respect to UMLS CUIs (extracted from 95.7% versus 94.5%), neuroimaging features (extracted from 94.1% versus 92.0%), cardiac features (95.4% versus 93.0%), clinical history (90.3% versus 91.5%), and laboratory features (90.0% versus 92.3%).

**Figure 2.**
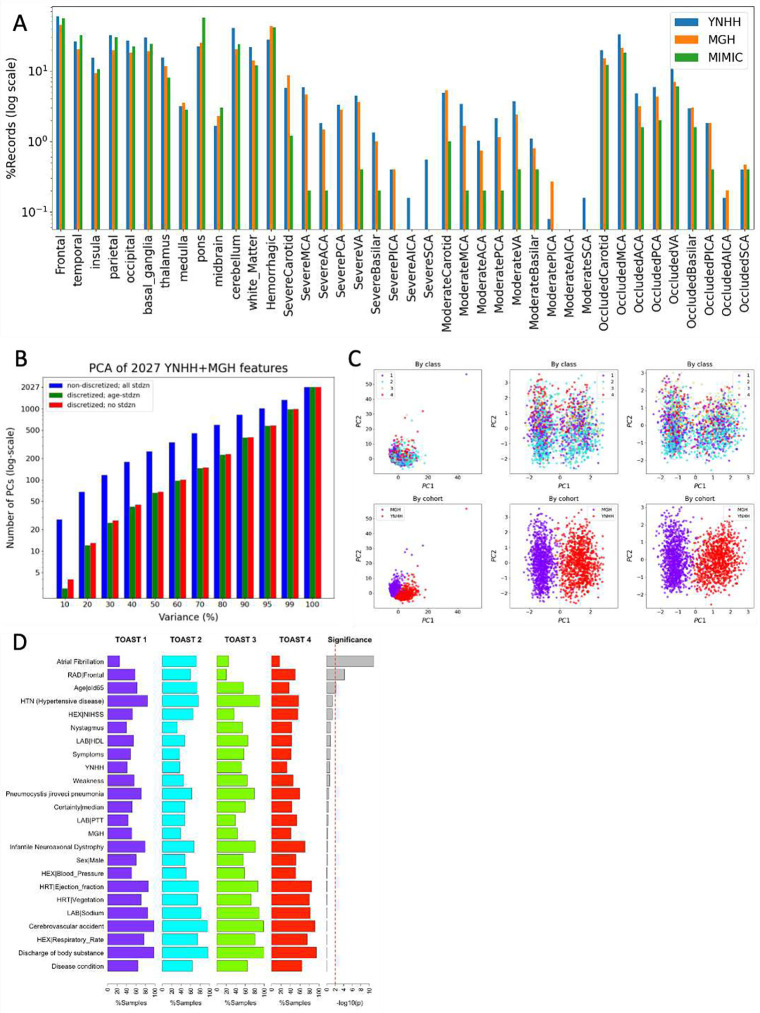
Exploratory data analysis. (A) Percentage comparison of discharge summary records with radiology-related features among the 3 cohorts. (B) Numbers of PCs for each PCA total variance cutoff for 2,027 YNHH and MGH features in the case of non-discretized features with all standardized continuous features, discretized features with the standardized age feature, and discretized features with no standardization. (C) Scatter plots of PC1 and PC2 for the three cases in (B) by class and by cohort. (D) Top features which are present in >50% of non-cryptogenic stroke records for each TOAST class and their significance by chi-squared tests.

Characteristics of the combined derivation cohort were compared with those of the external validation MIMIC-III cohort. The external validation cohort was comparable in age as the combined derivation cohort. Median word count of the external validation cohort discharge summaries was significantly lower (1712 words [IQR 1160–2294], p=0.002). The external validation cohort was more likely to have heart failure (27.3% versus 12.5%, p=0.019). The distribution of stroke etiologies differed significantly between the derivation and external validation cohorts (p=0.001). Large artery atherosclerosis (8.8% versus 20.5%, p=0.031) and small vessel disease (3.6% versus 12.8%, p=0.023) were significantly less frequent in the external validation cohort, while cardioembolism was significantly more frequent (51.2% versus 31.3%, p=0.028). The derivation and external validation cohorts were similar in terms of feature completeness (p=0.638–0.979) (Table 1; Figure 2A).

### Data Post-Processing and Principal Component Analysis

Of the 2,039 samples in the YNHH and MGH cohorts, a total of 1,932 samples were included for model derivation having successfully been post-processed by MetaMap. Imputation of missing entries in categorical and numerical features was performed using MICE in the derivation cohort and Random Forests-based imputation in the external validation cohort (**Table S3**). All subsequent analyses were performed on the imputed datasets.

For the non-cryptogenic stroke derivation cohort of 1,932 patients from YNHH and MGH analyzed for model development, we performed PCA on all of 2,027 features, either discretized or not, to reduce dimensionality or noise. We then selected the top PCs for each of the 10 thresholds of the total variance (see Methods) for model development. We found that 99% of the total variance could be explained by less than half of all features, the first principal component with about 4.5% variance discriminating between the two cohorts (Figures 2B and 2C).

### Base Models with Optimized Hyperparameters and Model Performances

**Figure 3.**
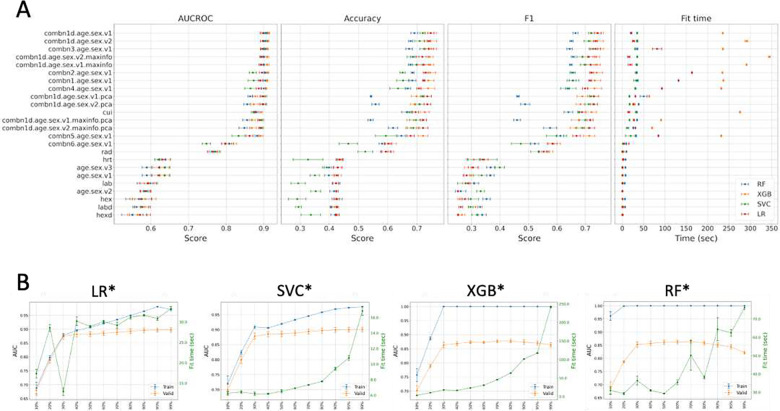
Model performances. (A) Performances and fit times of each optimized model for each feature group by 5-fold CV (mean +/− SD). (B) AUCROC and fit times of the PCA-based optimized models with combn1d.age.sex.v1.

We performed 96 hyperparameter optimizations (HPOs) for the 4 supervised machine-learning algorithms of LR, SVC, RF, and XGBoost and 24 training datasets (Tables 2A and **S4**; Figure 3A). Based on the AUCROC rankings in the 5-fold CV (**Table S5**), we denote the best model for each of the 4 strategies as LR*, SVC*, RF*, and XGB*, respectively, hereafter. All 4 best models were built using the full features with discretization (age + sex + CUI + RAD + HRT + HEXd + LABd, denoted by Λ_**1**_) (Table 2A). AUCROC and mean cross-validated accuracy were 89.8% and 74.7% for LR*, 90.1% and 71.9% for SVC*, 91.3% and 74.6% for XGB*, and 90.5% and 69.1% for RF*. Similar performances were observed with PCA of the full features (denoted by Λ_**1_pca**_), except for RF* (Table 2A). Fit times for XGB* with Λ_**1**_ were particularly longer (>235 sec) than those for the other 3 models (Table 2A). We also observe that XGB and RF tend to overfit (Figures 3B and S2). CUIs contributed most to model performance as measured by AUCROC, while the radiologic features ranked second. Decrease in performance was largest for each model when CUIs were excluded from the full feature group. On the other hand, excluding the LAB and HEX features tend to improve the performances. There was no performance improvement with those samples of high feature information defined by the presence of at least 4 features groups.

Next, we evaluated the performance of each optimized model for the full cohort of the 1,932 samples. We also built and examined SVC2 model, which calculates alternative prediction probabilities as a different calibration approach using the optimized hyperparameters from SVC* (Supplementary Information). The runtimes for the 5 models of LR*, SVC*, RF*, XGB*, and SVC2 were 114ms, 10.8s, 258ms, 475ms, and 10.8s, respectively, and their accuracies were 90.4%, 86.2%, 92.4%, 97.6%, and 88.1%. The numbers of samples correctly predicted by N = 1, 2, 3, 4, and 5 models (i.e., supports) are 59 (3.1%), 74 (3.8%), 92 (4.8%), 108 (5.6%), and 1574 (81.5%), respectively. In other words, 91.9% of all samples were correctly predicted by at least 3 models. The remaining 25 samples (1.3%) were incorrectly predicted by all the 5 models. The [numbers, percentages] of 1,002 MGH and 930 YNHH samples with N = 0 to 5 supports are [(13, 12), (1.3%, 1.3%)], [(32, 27), (3.2%, 2.9%)], [(31, 43), (3.1%, 4.6%)], [(44, 48), (4.4%, 5.2%)], [(57, 51), (5.7%, 5.5%)], and [(825, 749), (82.3%, 80.5%)], respectively. When we analyzed those 59 samples correctly predicted by a single model (N = 1), RF* was found to correctly predict 49 (83.1%) samples, in particular for TOAST 1 and 2 (22 and 16 samples or 37.3% and 27.1%, respectively).

### Performance of Ensemble Models and Consensus Meta-Model, StrokeClassifier

We aggregated the 4 optimized models built using the full features and samples, **X(Λ**_**1**_**)**, along with SVC2, into 4 ensemble models with 4 pre-specified summary statistics (see Methods). The 5-fold CV performance metrics associated with these ensemble models are shown in Table 2B. We observed performance improvement using the ensemble models by up to 0.7% on average (F1 score) in MEAN across the 7 metrics compared to the individual base models. No single ensemble model performed better than the rest in predicting each TOAST classification; there was variability among models which predicted each TOAST classification most accurately (**Tables S5-S7**). Spearman correlation and Cohen’s kappa values among the 9 base classifiers range from 0.78 and 0.81 (between RF* and SVC2) to 0.96 and 0.97 (between MEAN and MEDIAN), respectively. This observation supported our inclination to utilize a consensus ensemble meta-model, designated as *StrokeClassifier*, to harness the varying predictive capacities of the 9 classifiers while diluting the bias introduced by individual models, bolstering the robustness and generalizability of the model’s output.

*StrokeClassifier* demonstrated the following performance measures on average for predicting the 4-level outcome of non-cryptogenic stroke etiology: accuracy of 0.744, balanced accuracy of 0.710, weighted F1 of 0.740, and Cohen’s kappa of 0.629 (Table 2B), indicating substantial agreement with vascular neurologist-adjudicated stroke etiology. The mean accuracy of *StrokeClassifier* for each specific etiology versus not as a binary outcome ranged from 0.829 for TOAST 2 to 0.913 for TOAST 4 (Table 3).

### Performance Validation Using 300 Repeated Multi-Fold CV Splits

**Figure 4.**
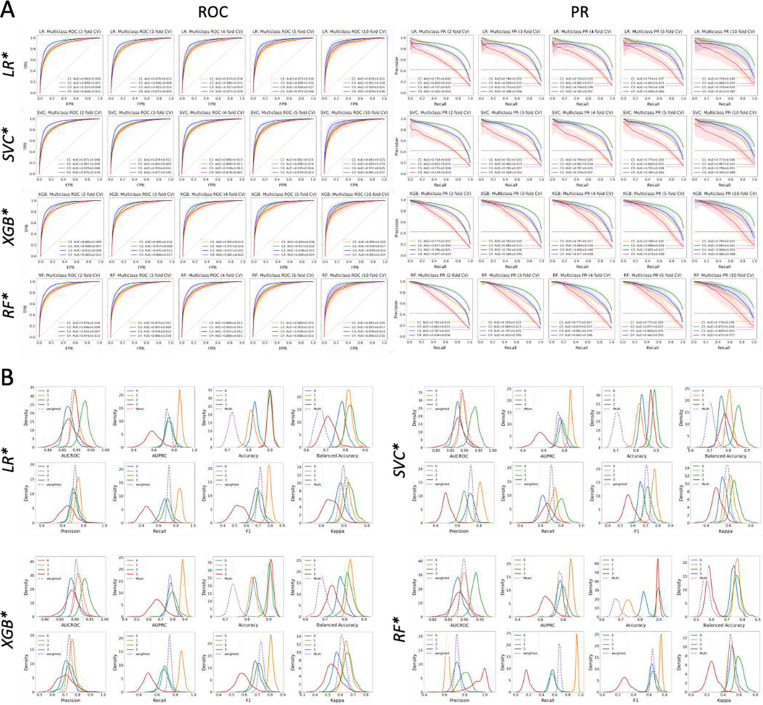
Model validation by RMFCV300. (A) ROC and PR curves for each optimized model and each CV fold by the RMFCV300 strategy. AUCROC and AUPRC are shown for each class vs. the rest. (B) Distributions of multiple performance metrics for each optimized model and each class (vs. the rest) as well as (weighted) averages.

Since cross-validation strategies such as the 5-fold CV used for HPO are anchored to a particular seed number, which is subjective, we used 300 training-validation data splits by repeated multi-fold CV, *RMFCV300*, to derive better estimates of model performance and generalization errors. We performed RMFCV300 for the 4 best models optimized by the HPO, focusing on model performances by AUCROC and AUPRC metrics (Figs. 4 and S3; **Tables S8-S10**). While there was variability in the magnitude of model performance measures for each TOAST class among the 4 models, all 4 models performed best in predicting TOAST 3 in terms of AUCROC, while they performed best in predicting TOAST 2 in terms of AUPRC, regardless of the number of CV folds employed. For each TOAST class, the means and standard deviations of both AUCROC and AUPRC for the CV fold repetitions consistently increased with the increasing CV folds across the 4 models.

### Analysis of Age-Sex Strata

To evaluate whether there was heterogeneity in model performances based on patient age and sex, we assessed model performances in age-sex subgroups using the RMFCV300 validation sets (Tables 4 and **S11-S14**). We observed that *StrokeClassifier* tended to perform worse in the stratum of Male/Age>=65, in particular for predicting TOAST 3 and 4 (lowest mean F1 of 64.6% and 36.3% across all strata, respectively). In contrast, *StrokeClassifier* performed better in the stratum of Female/Age<65, in particular for predicting TOAST 3 and 4 (highest mean F1 of 80.6% and 68.7% across the strata, respectively). We note that all mean performance values were greater than 60%, except F1 scores in TOAST 4 for the 4 strata of Male (51.4%±8.1%), Age>=65 (50.8%±10.4%), Male/Age>=65 (36.3%±16.9%), and Male/Age<65 (56.1%±8.9%).

### Feature Importance Analysis

**Figure 5.**
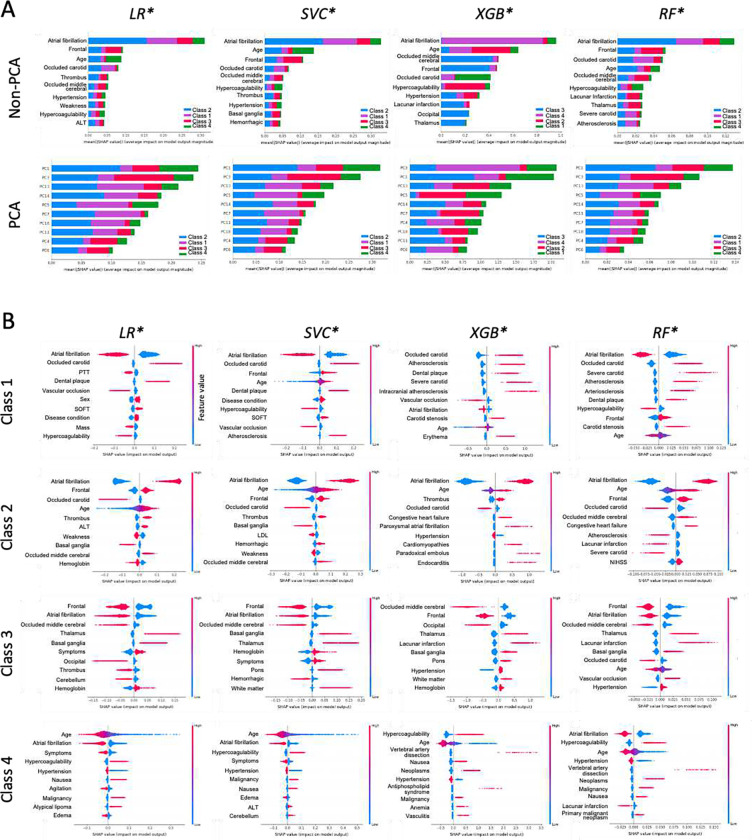
Feature importance by SHAP and statistical tests. (A) Top 10 features in terms of means of absolute SHAP values, mean(|SHAP|), across all classes for each optimized model for non-PCA-based and PCA-based feature groups. (B) Top 10 features (non-PCA) in terms of SHAP values for each class for each optimized model.

We examined feature importance or the contribution of features to predict TOAST classification by SHAP analysis for each of the 4 optimized base models. The top 10 features in terms of mean absolute SHAP values for each model are shown in Fig. 5A. The top feature for all 4 models is AF. The top second feature is either frontal location of infarct noted on radiography or patient age. For PCA, the top two features are PC1 and PC3 (the second and fourth principal components, respectively; 0-indexed). The largest impact of both AF and PC1 is on TOAST 2. We also examined the top 10 features for each class for each model as shown in Fig. 5B. The features that contribute the most to prediction of TOAST 1 by all models were AF, carotid occlusion, and atherosclerosis; for TOAST 2 were AF, patient age, and frontal location of infarct; for TOAST 3 were frontal location of infarct, occluded middle cerebral artery, AF, and thalamus location of infarct; and for TOAST 4, patient age, AF, and hypercoagulability or thrombophilia. For the PCA-based optimized models, we examined the top 5 PCs and the top 10 most contributing features for each PC for each class (Fig. S4; **Table S15**). Similar important features were observed including age, sex, and NIHSS. This method identified multiple unique features contributing to stroke etiology classes. For example, the following 6 features in PC11 were unique to TOAST 2 by 3 models (SVC*, XGB*, and RF*): blood pressure (HEX), mass of body region (C0577573), Macrophage Activation Syndrome (C1096155), cyclic neutropenia (C0221023), sinus (HRT), and hemorrhagic (RAD). The following 4 features in PC10 are unique to TOAST 3 by 3 models (LR*, SVC*, and XGB*): left ventricular hypertrophy (HRT; C0149721), pericardial effusion (C0031039), and agitation (C0085631). The top features by the model-agnostic Kolmogorov-Smirnov test and Student’s t-test are largely in agreement (Fig. S5 and Supplementary Information).

### Analysis of Misclassification

**Figure 6.**
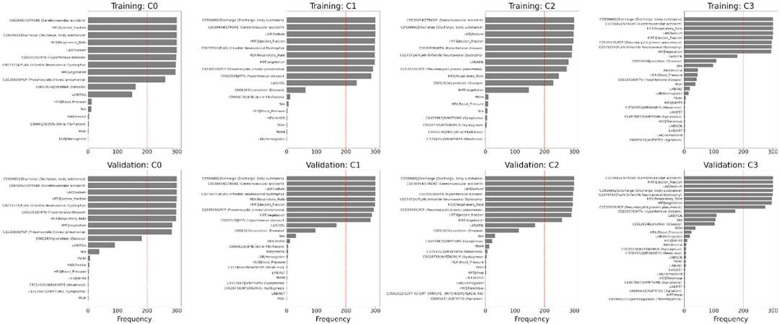
Top 10 features of misclassification. Top 10 features of misclassified samples for each class by the consensus model from RMFCV300.

We examined misclassified samples for each class and the top 10 features of the highest frequency among those misclassified samples. We analyzed classification results by *StrokeClassifier* for both training and validation from the merged RMFCV300 results. The misclassification or error rates (= 1 – accuracy; **Table S10**) for training were 4.5±0.6%, 5.3±0.7%, 2.5±0.4%, and 2.0±0.4% for the 4 classes, respectively, and those for validation were 16.2±1.4%, 16.8±1.7%, 9.4±1.2%, and 9.4±1.2% for the 4 classes, respectively. The top 10 most frequent features among misclassified samples for each class in each training or validation set are found to be present in >=54.8% of those samples (**Table S16**). Frequencies of those top 10 features in the 300 training or validation sets for each misclassified class are shown in Table 5 and Fig. 6. There are 6 features which are among the top 10 in all of the 300 training or validation sets: cerebrovascular accident, ejection fraction, body substance discharge, respiratory rate, sodium, and infantile neuroaxonal dystrophy.

### Model Generalizability by 5-way Cross-Hospital Validation

To test the model generalizability, we applied the 9 base models (with **X(Λ**_**1**_**)**) to the curated MIMIC discharge summaries (Table 6). We used 3 versions of the MIMIC data as external validation: (1) MIMIC^0^ = 375 non-cryptogenic samples with 1,406 features in common with YNHH and MGH, (2) MIMIC^1^ = 405 non-cryptogenic samples imputed by Random Forests using MICE, and (3) MIMIC^2^ = 405 non-cryptogenic samples imputed by random sampling using MICE. For MIMIC^1^, AUCROC ranged from 0.834 to 0.860 (0.847±0.009), accuracy from 0.667 to 0.711 (0.691±0.014), and F1 from 0.587 to 0.717 (0.690±0.039) by the 9 base classifiers, while *StrokeClassifier* showed AUCROC of 0.809, AUPRC 0.719, accuracy of 0.699, F1 of 0.708, and kappa 0.467 (Table 6A). Performances in MIMIC^0^ and MIMIC^2^ or those by the PCA-based models were similar (**Table S17**). Overall, the performance of *StrokeClassifier* in the external dataset was reduced by less than 5% in comparison with the internal 5-fold CV (Table 2B). We also examined class-wide performances of *StrokeClassifier* in MIMIC^1^. Prediction of TOAST 1 was associated with the lowest PPV of 37.0%, the lowest kappa of 0.377, and the highest false positive rate (FPR) of 11.4%; Prediction of TOAST 2 was associated with the lowest accuracy of 78.0%, the lowest F1 of 78.2%, the highest false negative rate (FNR) of 12.3%, the highest PPV of 84.1%, and the highest kappa of 0.535; Prediction of TOAST 3 was associated with the highest accuracy of 94.1%, the highest F1 of 94.6%, the lowest FPR of 4.0%, and the lowest FNR of 2.0%; performance measures for predicting TOAST 4 were moderate (Table 6B). Similar performances are observed for MIMIC^0^ and MIMIC^2^ (**Table S18**).

For an additional test of generalizability with **X(Λ**_**1**_**)**, we trained and optimized the 4 base models the same way as above using the MGH data of 1,002 non-cryptogenic samples and applied to the YNHH and MIMIC data for external validation (Tables 6B and **S18**). The 4 best models, LR*_MGH_, SVC*_MGH_, XGB*_MGH_, and RF*_MGH_, yielded mean cross-validated AUCROC of 91.0%, 90.9%, 92.3%, and 91.1%, respectively, and accuracy of 74.4%, 73.6%, 76.8%, and 68.1%, respectively. The external validation of the YNHH and MIMIC^1^ data by *StrokeClassifier* resulted in accuracy of 68.9% and 70.9%, respectively. Similarly, we next tested the models using the YNHH data of 930 non-cryptogenic samples for training and the MGH and MIMIC data for external validation (Tables 6B and **S18**). The 4 best models, LR*_YNHH_, SVC*_YNHH_, XGB*_YNHH_, and RF*_YNHH_, yielded mean cross-validated AUCROC of 86.8%, 86.5%, 87.6%, and 87.3%, respectively, and accuracy of 69.4%, 68.6%, 69.4%, and 60.6%, respectively. The external validation of the MGH and MIMIC^1^ data by *StrokeClassifier* resulted in accuracy of 70.3% and 66.4%, respectively. Performances in MIMIC^0^ and MIMIC^2^ were similar (**Table S18**).

### Predicting Etiologies of Cryptogenic Stroke Using StrokeClassifier

**Figure 7.**
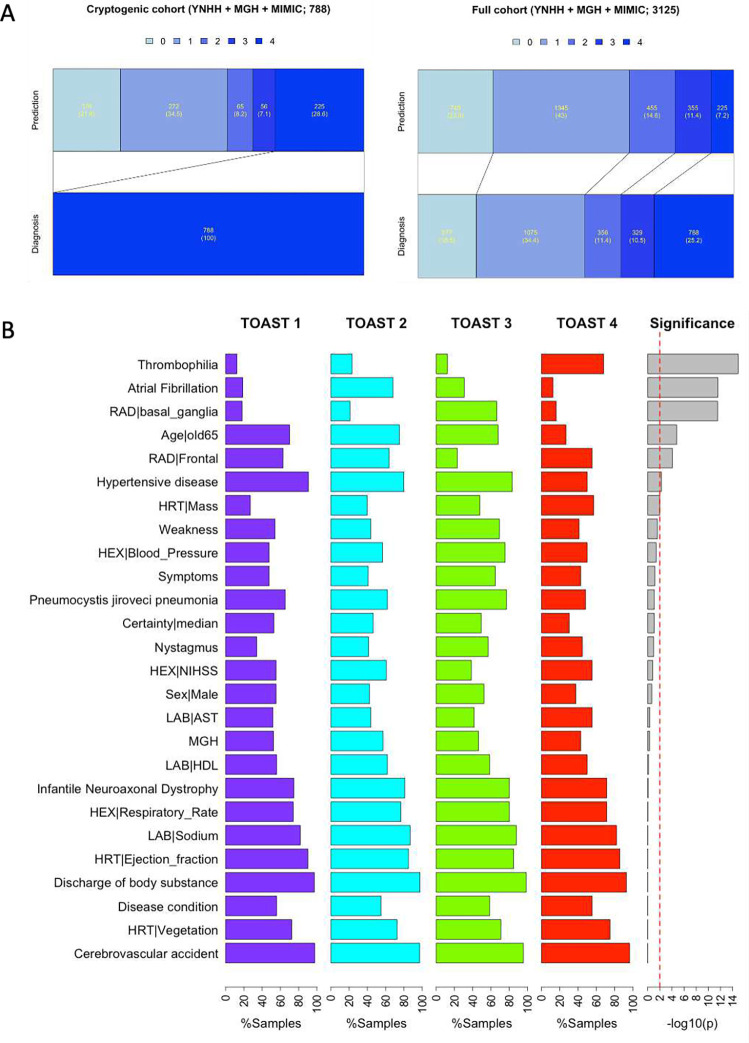
Prediction of cryptogenic samples and highly frequent features for each predicted class. (A) The bar graphs show a prediction distribution of all cryptogenic patients by StrokeClassifier (left) and a resultant prediction distribution of all of non-cryptogenic and cryptogenic patients (right). (B) The bar plots show class-wide frequency distributions of highly frequent features. There are 26 features which are present in >50% of those cryptogenic samples of any predicted TOAST class. The significance was tested by chi-squared tests.

We next aimed to classify a potential etiology of strokes in a cohort of adjudicated cryptogenic strokes using a variety of certainty heuristics as a proof-of-concept. In the pooled cohort of YNHH, MGH, and MIMIC^1^ datasets, there were a total of 788 stroke patients (285, 409, and 94, respectively), which were deemed to be cryptogenic strokes by vascular neurologists (Table 7). The heuristic that we employed in this study was built on a threshold of the first quartile (25% or moderate confidence) of the number of consensus supports among the 9 base classifiers for each TOAST classification based on the MIMIC^1^ external validation results: 7 supports for TOAST 1, 9 for TOAST 2, 7.2 for TOAST 3, and 7 for TOAST 4 (**Table S19**). If the number of supports for a particular sample was greater than or equal to the prespecified TOAST class threshold, the ischemic stroke was classified as the corresponding TOAST class. If the number of supports was less than any of the pre-specified TOAST class thresholds, the etiology was classified as persistently cryptogenic. Table 7 shows distributions of predicted TOAST classifications of cryptogenic patients for each cohort and the pooled cohort. Figure 7A also depicts the distributions of TOAST classification of the full cohort as adjudicated by vascular neurologists versus *StrokeClassifier*. Predictions for 46.3%, 54.5%, and 37.2% of the cryptogenic samples of YNHH, MGH, and MIMIC^1^ were agreed by all the 9 base classifiers, respectively. The prediction agreement by at least 8 base classifiers was observed for 69.8%, 72.6%, and 61.7% of the cryptogenic samples of YNHH, MGH, and MIMIC^1^, respectively. The most frequently predicted etiology was TOAST 2 for YNHH and MGH (32.6% and 37.9%, respectively) and TOAST 1 for MIMIC^1^ (27.7%), whereas the least frequently predicted etiology was TOAST 4 for YNHH and MGH (6.7% and 5.9%, respectively) and TOAST 3 for MIMIC^1^ (5.3%) (Table 7). The percentages of persistently cryptogenic samples for YNHH, MGH, and MIMIC^1^ were 30.9%, 27.1%, and 27.7%, respectively (Table 7). In other words, 28.6% of all cryptogenic samples (225 out of 788) were not predicted with high confidence by *StrokeClassifier* and remain cryptogenic. This reduced the percentage of cryptogenic patients from 25.2% to 7.2% in the full cohort of 3,125 stroke patients in YNHH, MGH, and MIMIC (Fig. 7A). In contrast, when we used a certainty heuristic of the third quartile number of consensus supports (high confidence), 9.9% of cryptogenic patients (309 cryptogenic patients of the full cohort; **Table S19**) remained persistently cryptogenic.

Finally, we generated a repertoire of EHR signatures of predicted TOAST classes for cryptogenic strokes (excluding the 225 persistently cryptogenic strokes) using feature frequencies from *StrokeClassifier*. We focused on those features which were present in >50% of the cryptogenic stroke samples in each predicted class. We identified 26 such features (Fig. 7B). Six of these 26 features were class-specific with p-value < 0.01 by chi-squared tests: hypercoagulability/thrombophilia (high-frequency for TOAST 4; p = 1.19e-15), AF (high-frequency for TOAST 2; p = 2.69e-12), basal ganglia (high-frequency for TOAST 3; p = 2.93e-12), age >65 (low-frequency for TOAST 4; p = 1.68e-05), frontal (low-frequency for TOAST 3; p = 8.60e-05), and hypertensive disease (low-frequency for TOAST 4; p = 5.66e-03).

## Discussion

We developed and validated a novel, accurate, and computationally efficient automated tool, *StrokeClassifier*, to predict AIS etiology using EHR text-based data collected during the stroke hospitalization. *StrokeClassifier* is a meta-classifier of a majority voting ensemble built from 9 base classifiers trained using adjudicated outcomes curated from institutions with vascular neurology expertise. Standardized CUI features extracted from unstructured or semi-structured text corpora by an NLP method were particularly powerful predictors. We found that the predictive capacity of *StrokeClassifier* was generalizable in 5-way external validation cohorts. The external validation accuracy of about 70% that we achieved was the minimum accuracy desired by a convenience sample of 13 international clinicians who care for stroke patients to adopt an AI stroke etiology diagnostic tool into clinical practice (8 vascular neurologists, 3 non-vascular neurologists, and 2 internists whom we interviewed during the National Science Foundation Innovation Corps Regional Program, Summer 2023). By applying *StrokeClassifier* to a cohort of cryptogenic stroke patients to predict non-cryptogenic stroke etiologies with a certainty heuristic, the proportion of ischemic stroke patients in the full cohort with a persistently cryptogenic diagnosis was 7.2%, which was 71% lower than the rate adjudicated by vascular neurologists. With further training in representative cohorts, *StrokeClassifier* may aide stroke etiology diagnosis during the stroke hospitalization and timely administration of secondary stroke prevention therapies. It may also inform future clinical and population research investigations.

There are three published manuscripts and one abstract describing machine learning classifiers for ischemic stroke TOAST classification subtyping with various limitations that we aimed to overcome^27,28,32^ . Inclusion criteria for specific stroke etiologies varied in these studies with downstream implications. The studies by Garg et al. and Turner et al. trained models to classify all 5 TOAST subtypes^27,32^, while the study by Wang et al. excluded cryptogenic strokes altogether^29^. Kamel et al. trained a binary classifier using non-cryptogenic stroke samples and then applied the classifier to cryptogenic stroke samples^28^. We utilized a stepwise approach, with a goal of ultimately classifying subtypes. We did not consider cryptogenic samples during training because they were comprised of a mixture of potential etiologies^58^. Instead, we investigated distributions of the 4 predicted non-cryptogenic etiologies for cryptogenic samples. We then developed various certainty heuristics to predict the probability of stroke etiologies, both non-cryptogenic and persistently cryptogenic. This scalable property of *StrokeClassifier* is promising since the patients it is tasked to classify will not be pre-specified as cryptogenic or non-cryptogenic. All published stroke etiology classifiers were trained and tested at a single center, which may not generalize to other centers in the U.S. or globally^27–29,32^. *StrokeClassifier* was tested in separate hospital cohorts with various EHR systems and robustness was demonstrated. Each classifier with the exception of the one developed by Garg et al. relied on hard-coded fields and did not have the capacity to utilize unstructured text data. Although the classifier generated by Garg et al. applied natural language processing to text-based data, it lacked an established ontological framework that can map phraseologies to consistent clinical concepts. We leveraged the UMLS conceptual framework developed by the National Library of Medicine to ensure the operability of *StrokeClassifier* irrespective of clinician and computer environment. For computational efficiency, we utilized PCA to capture multi-dimensional contributions of a wide array of features. We uniquely trained *StrokeClassifier* on adjudicated stroke etiologies upon review by at least two board-certified vascular neurologists. Since there was variability among individual optimized models in predicting each etiology, the 4 optimized models along with SVC2 were aggregated into ensemble models, which are also architecturally simple and efficient. Although ensemble modeling was utilized by Kamel et al.^28^, it did not include the diversity of models that *StrokeClassifier’s* meta-model represents with summary-statistic based ensemble models. We took several measures to minimize bias. To address overfitting, we investigated sub-optimal models within 1 standard deviation of the optimized models in terms of AUCROC, showing performance reduction by up to 4% across different metrics and CV folds. Additionally, in an effort to offset bias introduced by relying on a single choice of CV folds and a particular random seed, our RMFCV300 strategy analysis offers a more robust framework to assess model performance and generalization errors. Finally, we performed SHAP analyses to assess the degrees to which features contributed to stroke etiology prediction. The features contributing to the prediction of each stroke etiology were biologically plausible, lending validity to *StrokeClassifier*.

There are multiple potential applications of a trained, automated, accurate, and computationally efficient stroke etiology classifier. It can be implemented in health systems to perform the complex task of synthesizing the copious, semi-structured data collected during an AIS hospitalization and rapidly classifying the underlying stroke etiology in an automated manner for millions of patients. Most proximally, automated stroke etiology prediction can cue a treating clinician to consider instituting a targeted treatment by reducing diagnostic uncertainty, diagnostic errors due to human cognitive biases, oversight, and therapeutic inertia^59^. In healthcare settings where vascular neurology expertise is sparse or unavailable, *StrokeClassifier* may be especially valuable^23^. A classifier such as *StrokeClassifier* can be harnessed by informaticians to create nudges or progress notes indicating predicted etiologies and guideline-recommended therapies for individual patients. Stroke etiology data fields collected by manual extraction are currently incomplete in registries in the U.S. at all levels, and when populated, are often inaccurate as seen in our study. Stroke etiology predictions can be linked to institutional, regional, and country-wide registries to facilitate quality improvement, clinical trials, public health, and health services research efforts. Finally, it may identify patients with established stroke etiologies and risk factors which may render them eligible for clinical trials studying novel secondary stroke prevention therapies.

The capacity to predict an underlying etiology of cryptogenic strokes using *StrokeClassifier* is promising. The predicted etiology among cryptogenic patients in the YNHH and MGH cohorts was predominantly cardioembolism, varying from 33% to 38%, followed by large artery atherosclerosis in 19% to 22%. Secondary analysis of the NAVIGATE ESUS study demonstrated that among ESUS patients, there were multiple potential etiologies including atrial cardiopathy (37%), left ventricular disease (36%), and arterial atherosclerosis (29%), with no potential etiology found in only 23% of patients and more than 1 potential etiology in 41% of patients^58^. Given that many cryptogenic stroke patients have multiple potential sources, applying an algorithm such as *StrokeClassifier* can be especially fruitful because its supervised learning of features that may non-linearly associate with etiologies may be transferable. We derived EHR signatures corresponding to the predicted etiology of cryptogenic stroke patients. It begins to provide a conceptual and workflow framework for strokes traditionally deemed cryptogenic. For instance, cryptogenic patients with predicted etiology of large artery atherosclerosis by *StrokeClassifier* tend to be older and have frontal infarct, hypertension, and no AF. Thus, predicted stroke etiology classification of patients with these features during stroke hospitalization may prompt deeper, streamlined inquiry into this potential mechanism such as more advanced vascular imaging to assess the characteristics of a sub-stenotic carotid plaque. It may also obviate the need for broad, unnecessary testing that leads to health care expenditure. Predictions may also tip clinicians uncertain about which of multiple competing etiologies led to the stroke into a singular direction. This information and subsequent diagnostic investigation may then lead to initiation of evidence-based targeted secondary stroke prevention therapy. Finally, in an era of biomarker-based clinical studies, the potential stroke etiology signatures yielded by classifiers such as *StrokeClassifier* may advance research by identifying an enriched population of cryptogenic ischemic stroke patients who may benefit from specific trial interventions for secondary stroke prevention.

Our study has limitations. The scope of this study was limited by its cross-sectional design; our future goal is further training *StrokeClassifier* in longitudinal cohorts to enable it to predict the eventual etiologic diagnosis in patients initially deemed cryptogenic. While the gold standard method of discerning stroke etiology is based on pathologic confirmation, an invasive procedure such as a brain biopsy is exceedingly rare. Thus, our outcome measure, while adjudicated by vascular neurology specialists, is ultimately probabilistic. Although training occurred using data from two academic institutions which are Comprehensive Stroke Centers, there was notable variability in clinical documentation and degree of testing by site as well as in prediction performances (Tables 1 and 6B). Nevertheless, training *StrokeClassifier* in this heterogeneous environment ensured generalizability across clinician training and documentation styles, EHR systems, and formatting. Further training in other cohorts is needed to increase capture of more features. The epidemiology of stroke etiology may differ by geographic region, race, or ethnicity, and prevalence may impact predictive accuracy^60^. Finally, despite the identification of optimal models via HPO, there remains room for further exploration of other hyperparameters.

In conclusion, we present *StrokeClassifier*, a validated diagnostic tool developed using an innovative modeling strategy which allows automated, real-time classification of stroke etiology in an accurate and computationally efficient manner with EHR text data inputs. Its immediate application may be as a clinical decision support tool to aide in the diagnosis of stroke etiology, prompting targeted secondary stroke prevention therapies in a timely manner. Furthermore, *StrokeClassifier* may facilitate abstraction of stroke etiology in population-based registries to aide epidemiologic, health policy, and clinical research efforts.

## Supplementary Material

Supplement 1

## Figures and Tables

**Table 1.** Description of study cohorts

	Data for model development			Data for external validation	P-value	
	YNHH (N=1269)	MGH (N=1493)	YNHH + MGH (N=2762)	MIMIC from BIDMC (N=500)	YNHH vs. MGH	YNHH+MGH vs. MIMIC
Age (median [IQR Q1-Q3])	71 [59–82]	69 [59–79]	70 [59–80]	73 [61–82]	0.01295	0.45335
Male sex	636 (50.1%)	812 (54.4%)	1448 (52.4%)	232 (46.4%)	1	1
Admission Year	2015–2020	2016–2019	2015–2020	2001–2012	NA	NA
Characters in discharge summary texts (median [IQR Q1-Q3])	11294 [8865–14033]	13338 [10366–16508]	12255 [9530–15590]	11436 [7650–15184]	2.57E-18	0.00457
Words in discharge summary texts (median [IQR Q1-Q3])	1639 [1274–2064]	2058 [1593–2554]	1846 [1410–2365]	1712 [1160–2294]	1.21E-35	0.00214
NIHSS (median [IQR Q1-Q3]; %N)	5 [1–11]; 68.7%	6 [2–15]; 34.3%	5 [2–13]; 50.1%	16 [10–20]; 9.2%	NA	NA
Co-morbidities (CUI freq.)					0.00721	0.31430
Hypertension	1006 (82.1%)	1118 (78.5%)	2124 (80.2%)	384 (77.0%)	0.77635	0.79867
Hyperlipidemia	403 (32.9%)	164 (11.5%)	567 (21.4%)	98 (19.6%)	0.00132	0.78388
Diabetes	571 (46.6%)	505 (35.4%)	1076 (40.6%)	214 (42.9%)	0.21615	0.80278
Atrial fibrillation	476 (38.9%)	739 (51.9%)	1215 (45.8%)	215 (43.1%)	0.17248	0.76954
Cigarette use	1 (0.1%)	0 (0%)	1 (0.04%)	0 (0%)	0.75183	0.84597
Drug use	14 (1.1%)	32 (2.2%)	46 (1.7%)	10 (2.0%)	0.54483	0.88972
Coronary artery disease	218 (17.8%)	57 (4.0%)	275 (10.4%)	104 (20.8%)	0.00312	0.06109
Heart failure	174 (14.2%)	157 (11.0%)	331 (12.5%)	136 (27.3%)	0.52383	0.01919
Stroke Etiology					0.80070	0.00103
Large artery atherosclerosis (1)	251 (19.8%)	314 (21.0%)	565 (20.5%)	44 (8.8%)	0.84461	0.03066
Cardioembolism (2)	418 (32.9%)	446 (29.9%)	864 (31.3%)	256 (51.2%)	0.69881	0.02846
Small vessel disease (3)	194 (15.3%)	160 (10.7%)	354 (12.8%)	18 (3.6%)	0.37006	0.02310
Other determined (4)	113 (8.9%)	143 (9.6%)	256 (9.3%)	88 (17.6%)	0.87554	0.10953
Cryptogenic (5)	293 (23.1%)	430 (28.8%)	723 (26.2%)	94 (18.8%)	0.42780	0.26997
Degree of Feature Completeness					0.99900	0.97650
UMLS CUIs (CUI)	1215 (95.7%)	1425 (94.5%)	2626 (95.1%)	499 (99.8%)	0.92854	0.73506
Neuroimaging (RAD)	1194 (94.1%)	1373 (92.0%)	2567 (92.9%)	484 (96.8%)	0.87606	0.77930
Cardiac data (HRT)	1210 (95.4%)	1389 (93.0%)	2599 (94.1%)	492 (98.4%)	0.86597	0.75654
Clinical History (HEX)	1146 (90.3%)	1366 (91.5%)	2512 (90.9%)	453 (90.6%)	0.92989	0.97936
Laboratory data (LAB)	1142 (90.0%)	1378 (92.3%)	2520 (91.2%)	425 (85.0%)	0.86443	0.63842
MetaMap						
Processing time on average (min)	5.0	3.3	4.1	0.8		

Chi-squared tests for categorical variables and Student’s t-tests for numerical variables. Those p-values < 0.05 are highlighted in red.

**Table 2.** Optimized model performances

(A) Validation results of the optimized base models for each feature group																
Feature groups	Fit time (sec)				AUCROC				Accuracy				F1			

	LR*	SVC*	XGB*	RF*	LR*	SVC*	XGB*	RF*	LR*	SVC*	XGB*	RF*	LR*	SVC*	XGB*	RF*

age.sex.v1	0.314	0.717	1.358	7.755	0.620	0.637	0.636	0.589	0.425	0.350	0.427	0.398	0.283	0.335	0.300	0.366
	
age.sex.v2	0.111	0.370	0.408	0.761	0.585	0.589	0.586	0.583	0.424	0.353	0.424	0.417	0.252	0.332	0.252	0.262
	
age.sex.v3	0.109	0.773	1.341	7.471	0.622	0.637	0.636	0.589	0.429	0.391	0.427	0.399	0.305	0.399	0.300	0.367
	
hex	0.720	0.869	1.051	6.314	0.573	0.576	0.585	0.568	0.425	0.290	0.421	0.427	0.276	0.297	0.257	0.353
	
hexd	0.114	0.829	1.786	0.954	0.571	0.570	0.565	0.550	0.423	0.337	0.427	0.420	0.252	0.319	0.266	0.337
	
lab	0.226	1.338	2.033	6.001	0.593	0.591	0.596	0.599	0.422	0.294	0.422	0.423	0.287	0.279	0.290	0.308
	
labd	0.122	1.464	3.586	6.693	0.578	0.575	0.584	0.562	0.426	0.295	0.417	0.407	0.262	0.297	0.314	0.330
	
hrt	2.148	1.369	3.486	7.686	0.631	0.619	0.631	0.638	0.435	0.327	0.439	0.435	0.343	0.338	0.307	0.308
	
rad	0.110	1.030	3.709	9.723	0.764	0.769	0.775	0.771	0.593	0.523	0.600	0.593	0.554	0.535	0.566	0.554
	
cui	15.291	32.001	275.934	34.236	0.876	0.872	0.892	0.880	0.695	0.674	0.727	0.665	0.688	0.677	0.722	0.640
	
combn1.age.sex.v1	131.999	32.179	236.997	35.784	0.891	0.873	0.908	0.904	0.724	0.652	0.739	0.684	0.716	0.656	0.733	0.655
	
combn2.age.sex.v1	163.475	32.328	234.616	34.418	0.895	0.871	0.911	***0.905***	0.730	0.651	0.745	0.685	0.722	0.654	0.739	0.657
	
combn3.age.sex.v1	81.605	30.247	235.918	35.326	0.896	0.898	0.910	***0.905***	0.740	***0.725***	***0.748***	0.673	0.735	***0.727***	***0.743***	0.644
	
combn4.age.sex.v1	92.858	30.702	232.069	31.381	0.887	0.865	0.906	0.900	0.706	0.635	0.737	0.669	0.699	0.640	0.732	0.637
	
combn5.age.sex.v1	84.851	19.786	231.799	32.263	0.862	0.833	0.892	0.882	0.678	0.593	0.720	0.647	0.669	0.598	0.713	0.612
	
combn6.age.sex.v1	4.455	1.621	15.531	9.306	0.798	0.747	0.815	0.808	0.602	0.465	0.608	0.580	0.584	0.473	0.589	0.522
	
***combn1d.age.sex.v1***	***21.406***	***35.117***	235.597	***19.809***	***0.898***	***0.901***	***0.913***	***0.905***	***0.747***	0.719	0.746	***0.691***	***0.744***	0.721	0.741	***0.665***
	
combn1d.age.sex.v2	26.649	35.415	290.860	34.993	***0.898***	0.900	0.912	0.904	0.741	0.709	0.746	0.679	0.735	0.712	0.741	0.651
	
combn1d.age.sex.v1.maxinfo	18.608	34.546	291.177	33.070	0.893	0.894	0.908	0.901	0.723	0.698	0.740	0.677	0.718	0.700	0.735	0.649
	
combn1d.age.sex.v2.maxinfo	15.444	35.102	344.686	35.421	0.893	0.894	0.908	0.901	0.718	0.686	0.743	0.685	0.712	0.689	0.738	0.657
	
combn1d.age.sex.v1.pca	30.833	16.843	63.394	50.176	0.897	0.900	0.878	0.864	0.735	0.718	0.698	0.543	0.731	0.720	0.689	0.463
	
combn1d.age.sex.v2.pca	26.028	17.572	29.503	38.544	0.897	0.899	0.873	0.856	0.735	0.710	0.691	0.558	0.730	0.712	0.682	0.488
	
combn1d.age.sex.v1.maxinfo.pca	27.267	17.190	69.948	34.900	0.890	0.893	0.868	0.856	0.722	0.696	0.679	0.541	0.717	0.699	0.666	0.460
	
combn1d.age.sex.v2.maxinfo.pca	30.783	12.510	90.964	25.857	0.891	0.894	0.869	0.848	0.721	0.687	0.689	0.618	0.714	0.690	0.676	0.576

(B) Validation results of the ensemble/meta models using combn1d.age.sex.v1 (X(Λ_1_))							
Model	AUCROC	AUPRC	ACC	BA	PRC	F1	KAPP
LR*	0.898±0.008	0.796±0.012	0.747±0.012	0.706±0.021	0.747±0.011	0.744±0.013	0.632±0.019
SVC*	0.900±0.009	0.802±0.013	0.728±0.005	0.694±0.010	0.726±0.007	0.725±0.005	0.606±0.007
SVC2	0.887±0.009	0.772±0.014	0.719±0.010	0.726±0.007	0.733±0.011	0.721±0.011	0.606±0.014
XGB*	0.913±0.003	0.827±0.014	0.746±0.023	0.697±0.037	0.745±0.026	0.741±0.025	0.627±0.035
RF*	0.905±0.005	0.817±0.010	0.691±0.013	0.582±0.012	0.738±0.016	0.665±0.010	0.523±0.021
**MAX**	0.907±0.006	0.821±0.012	0.736±0.009	**0.729±0.005**	0.740±0.010	0.736±0.010	0.626±0.012
**MIN**	0.907±0.007	0.819±0.011	0.743±0.009	0.709±0.015	0.743±0.011	0.740±0.010	0.628±0.014
**MEAN**	**0.912±0.005**	**0.826±0.009**	**0.750±0.008**	0.725±0.008	**0.749±0.009**	**0.748±0.009**	**0.640±0.011**
**MEDIAN**	0.910±0.005	0.823±0.008	0.749±0.010	0.715±0.012	0.748±0.012	0.745±0.011	0.636±0.014
***StrokeClassifier***	NA	NA	***0.744±0.009***	***0.710±0.015***	***0.743±0.009***	***0.741±0.010***	***0.629±0.014***

Those with MaxInfo ≥ 4 are denoted by a suffix of “.maxinfo” in the feature group names. Green: fit time < 20 sec, AUCROC ≥ 0.8, and Accuracy or F1 ≥ 0.7; Blue: 20 sec ≤ fit time < 200 sec, 0.6 ≤ AUCROC < 0.8, and 0.5 ≤ Accuracy or F1 < 0.7; Red: fit time ≥ 200 sec, AUCROC < 0.6, and Accuracy or F1 < 0.5.

N.B. mean ± s.d. for the 5 validation sets of 5-fold CV (seed = 1701). The highest mean value for each performance metric is highlighted in green in bold.

**Table 3.** Performance of *StrokeClassifier* for each TOAST classification

Physician Diagnosis	Accuracy	BA	PPV	F1	Kappa	FPR	FNR
Large artery atherosclerosis (1)	0.836±0.015	0.785±0.033	0.718±0.029	0.834±0.017	0.580±0.049	0.073±0.015	**0.091±0.021**
Cardioembolism (2)	**0.829±0.014**	0.830±0.011	**0.781±0.027**	**0.830±0.013**	0.654±0.025	**0.100±0.018**	0.071±0.007
Small vessel disease (3)	0.909±0.010	**0.854±0.010**	0.733±0.049	0.910±0.008	**0.693±0.024**	0.050±0.015	**0.041±0.006**
Other determined (4)	**0.913±0.006**	**0.764±0.037**	**0.685±0.038**	**0.909±0.008**	**0.568±0.046**	**0.033±0.010**	0.054±0.010

N.B. mean ± s.d. for 5 validation sets of 5-fold CV (seed = 1701).

**Table 4.** Performance of *StrokeClassifier* in age-sex strata

**Performance metric**	**TOAST**	**Female**	**Male**	**Age ≥ 65**	**Age < 65**	**Female, Age ≥ 65**	**Female, Age < 65**	**Male, Age ≥ 65**	**Male, Age < 65**
		*246.8±120.9*	*287.7±140.8*	*330.6±161.7*	*203.9±100.0*	*169.0±83.0*	*77.7±38.5*	*161.6±79.4*	*126.2±62.1*
**Accuracy**	1	0.862±0.023	0.817±0.019	0.837±0.020	0.839±0.026	0.856±0.027	**0.874±0.040**	**0.817±0.030**	0.818±0.032
	2	0.848±0.023	0.818±0.023	0.834±0.024	0.828±0.027	**0.855±0.029**	0.833±0.040	**0.812±0.036**	0.824±0.033
	3	0.919±0.017	0.894±0.017	0.901±0.016	0.914±0.019	**0.920±0.021**	0.916±0.032	**0.880±0.025**	0.912±0.028
	4	0.918±0.018	0.896±0.018	0.947±0.012	0.839±0.027	**0.956±0.017**	**0.835±0.040**	0.938±0.019	0.842±0.036
**Balanced Accuracy**	1	0.780±0.039	0.782±0.025	0.782±0.026	0.786±0.037	**0.772±0.043**	**0.797±0.069**	0.785±0.036	0.778±0.039
	2	**0.850±0.023**	0.813±0.024	0.835±0.024	0.796±0.034	0.848±0.030	0.810±0.050	0.817±0.035	**0.791±0.044**
	3	0.862±0.034	0.839±0.032	0.820±0.034	0.887±0.029	0.847±0.046	0.883±0.051	**0.794±0.049**	**0.891±0.042**
	4	**0.798±0.046**	0.711±0.048	0.692±0.052	0.764±0.040	0.757±0.085	0.793±0.054	**0.628±0.074**	0.739±0.060
**F1**	1	0.674±0.060	0.701±0.037	0.690±0.039	0.695±0.054	**0.661±0.072**	0.697±0.108	**0.707±0.049**	0.691±0.057
	2	0.844±0.024	0.771±0.030	0.841±0.023	0.718±0.048	**0.873±0.026**	**0.712±0.079**	0.797±0.040	0.718±0.065
	3	0.765±0.051	0.715±0.048	0.691±0.051	0.795±0.045	0.733±0.072	**0.806±0.086**	**0.646±0.076**	0.783±0.073
	4	0.669±0.073	0.514±0.081	0.508±0.104	0.624±0.063	0.610±0.146	**0.687±0.093**	**0.363±0.169**	0.561±0.089

N.B. mean ± S.D. of performance metrics from the RMFCV300 validation sets. The numbers in italic below each stratum name are mean ± s.d. of the sample sizes. For each TOAST for each metric, the largest mean value across the strata is highlighted in bold in green and the smallest mean value in bold in red. All mean values less than 0.5 are highlighted in bold italic in red.

**Table 5.** Top 10 features of the highest frequency for misclassification by *StrokeClassifier*

Misclassified Etiology	Training		Validation	
	Top 10 most frequent features	Frequency	Top 10 most frequent features	Frequency
**Large artery atherosclerosis (1)**	**C0038454|STROKE (Cerebrovascular accident)**	**300**	**C2926602|Discharge (Discharge, body substance)**	**300**
	**HRT|Ejection_fraction**	**300**	**C0038454|STROKE (Cerebrovascular accident)**	**300**
	**C2926602|Discharge (Discharge, body substance)**	**300**	**LAB|Sodium**	**300**
	**HEX|Respiratory_Rate**	**300**	**HRT|Ejection_fraction**	**300**
	**LAB|Sodium**	**300**	**C0270724|PLAN (Infantile Neuroaxonal Dystrophy)**	**299**
	**C0020538|HTN (Hypertensive disease)**	**299**	**C0020538|HTN (Hypertensive disease)**	**299**
	**C0270724|PLAN (Infantile Neuroaxonal Dystrophy)**	**297**	**HEX|Respiratory_Rate**	**298**
	**HRT|Vegetation**	**296**	**HRT|Vegetation**	**285**
	**C1535939|PCP (Pneumocystis jiroveci pneumonia)**	**261**	**C1535939|PCP (Pneumocystis jiroveci pneumonia)**	**283**
	C0012634|condition (Disease)	162	C0012634|condition (Disease)	182
	LAB|HDL	150	LAB|HDL	91
	HEX|Blood_Pressure	13	Sex	39
	Sex	12	YNHH	7
	RAD|Frontal	4	RAD|Frontal	5
	C0004238|AFib (Atrial Fibrillation)	3	HEX|Blood_Pressure	3
	MGH	2	HEX|NIHSS	3
	LAB|Hemoglobin	1	C3714552|WEAKNESS (Weakness)	2
			C1457887|SYMPTOMS (Symptoms)	2
			MGH	2
**Cardioembolism (2)**	**C2926602|Discharge (Discharge, body substance)**	**300**	**C2926602|Discharge (Discharge, body substance)**	**300**
	**C0038454|STROKE (Cerebrovascular accident)**	**300**	**C0038454|STROKE (Cerebrovascular accident)**	**300**
	**LAB|Sodium**	**300**	**LAB|Sodium**	**300**
	**HRT|Ejection_fraction**	**299**	**C0270724|PLAN (Infantile Neuroaxonal Dystrophy)**	**300**
	**C0270724|PLAN (Infantile Neuroaxonal Dystrophy)**	**298**	**HEX|Respiratory_Rate**	**299**
	**HEX|Respiratory Rate**	**298**	**HRT|Ejection_fraction**	**299**
	**HRT|Vegetation**	**298**	**C1535939|PCP (Pneumocystis jiroveci pneumonia)**	**294**
	**C1535939|PCP (Pneumocystis jiroveci pneumonia)**	**292**	**HRT|Vegetation**	**288**
	**C0020538|HTN (Hypertensive disease)**	**286**	**C0020538|HTN (Hypertensive disease)**	**284**
	**LAB|HDL**	**236**	LAB|HDL	169
	C0012634|condition (Disease)	64	C0012634|condition (Disease)	98
	C0004238|AFib (Atrial Fibrillation)	12	Sex	32
	Sex	7	HEX|NIHSS	11
	HEX|Blood_Pressure	3	C0004238|AFib (Atrial Fibrillation)	5
	HEX|NIHSS	2	RAD|Frontal	5
	MGH	2	LAB|Hemoglobin	4
	YNHH	2	HEX|Blood_Pressure	2
	LAB|Hemoglobin	1	C3714552|WEAKNESS (Weakness)	2
			LAB|ALT	2
			YNHH	2
			C1457887|SYMPTOMS (Symptoms)	1
			C0028738|NYSTAGMUS (Nystagmus)	1
			LAB|AST	1
			MGH	1
**Small vessel disease (3)**	**C0038454|STROKE (Cerebrovascular accident)**	**300**	**C2926602|Discharge (Discharge, body substance)**	**300**
	**C2926602|Discharge (Discharge, body substance)**	**300**	**C0038454|STROKE (Cerebrovascular accident)**	**300**
	**LAB|Sodium**	**298**	**LAB|Sodium**	**299**
	**HRT|Ejection_fraction**	**297**	**C0020538|HTN (Hypertensive disease)**	**296**
	**C0020538|HTN (Hypertensive disease)**	**294**	**C0270724|PLAN (Infantile Neuroaxonal Dystrophy)**	**295**
	**C0270724|PLAN (Infantile Neuroaxonal Dystrophy)**	**292**	**HEX|Respiratory_Rate**	**295**
	**LAB|HDL**	**281**	**C1535939|PCP (Pneumocystis jiroveci pneumonia)**	**293**
	**C1535939|PCP (Pneumocystis jiroveci pneumonia)**	**275**	**HRT|Ejection_fraction**	**291**
	**HEX|Respiratory_Rate**	**248**	**HRT|Vegetation**	**259**
	**C0012634|condition (Disease)**	**230**	LAB|HDL	168
	HRT|Vegetation	147	C0012634|condition (Disease)	113
	YNHH	10	Sex	33
	HEX|Blood_Pressure	9	C1457887|SYMPTOMS (Symptoms)	23
	Sex	7	YNHH	13
	C1457887|SYMPTOMS (Symptoms)	6	C3714552|WEAKNESS (Weakness)	7
	C0028738|NYSTAGMUS (Nystagmus)	4	C0028738|NYSTAGMUS (Nystagmus)	4
	C0004238|AFib (Atrial Fibrillation)	1	HEX|Blood Pressure	2
	C3714552|WEAKNESS (Weakness)	1	MGH	2
			HRT|sinus	2
			HEX|NIHSS	1
			LAB|Hemoglobin	1
			HRT|Thrombus	1
			C3542022|SOFT	1
			C0085631|AGITATED (Agitation)	1
			C0085631|AGITATED (Agitation)	1
**Other determined (4)**	**C2926602|Discharge (Discharge, body substance)**	**300**	**C0038454|STROKE (Cerebrovascular accident)**	**300**
	**C0038454|STROKE (Cerebrovascular accident)**	**300**	**HRT|Ejection_fraction**	**300**
	**HEX|Respiratory_Rate**	**300**	**LAB|Sodium**	**300**
	**LAB|Sodium**	**300**	**C2926602|Discharge (Discharge, body substance)**	**300**
	**HRT|Ejection_fraction**	**299**	**C0270724|PLAN (Infantile Neuroaxonal Dystrophy)**	**299**
	**C1535939|PCP (Pneumocystis jiroveci pneumonia)**	**295**	**HEX|Respiratory_Rate**	**297**
	**C0270724|PLAN (Infantile Neuroaxonal Dystrophy)**	**295**	**HRT|Vegetation**	**295**
	**HRT|Vegetation**	**294**	**C1535939|PCP (Pneumocystis jiroveci pneumonia)**	**274**
	LAB|HDL	180	C0020538|HTN (Hypertensive disease)	173
	C0012634|condition (Disease)	108	LAB|HDL	110
	Sex	98	Sex	106
	RAD|Frontal	46	C0012634|condition (Disease)	103
	HEX|Blood_Pressure	45	MGH	38
	C0020538|HTN (Hypertensive disease)	43	HEX|Blood_Pressure	25
	MGH	38	LAB|Hemoglobin	19
	LAB ALT	19	HEX|NIHSS	11
	LAB|Hemoglobin	15	RAD|Frontal	7
	YNHH	5	C0028738|NYSTAGMUS (Nystagmus)	6
	HEX|NIHSS	4	C3714552|WEAKNESS (Weakness)	6
	C3714552|WEAKNESS (Weakness)	3	LAB|LDL	6
	LAB|PTT	3	YNHH	5
	C1457887|SYMPTOMS (Symptoms)	2	LAB|ALT	5
	HRT|Thrombus	2	LAB|AST	4
	LAB|LDL	2	LAB|Hematocrit	2
	LAB|AST	2	HRT|Thrombus	2
	LAB|Hematocrit	1	C1457887|SYMPTOMS (Symptoms)	2
	C0085631|AGITATED (Agitation)	1	LAB|PTT	2
			C0085631|AGITATED (Agitation)	1
			HRT|Mass	1
			C0398623|Hypercoagulable (Thrombophilia)	1

**Table 6.** Model generalizability

(A) Global performances (weighted averages over all classes) on MIMIC by individual models							
Model	AUCROC	AUPRC	ACC	BA	PRC	F1	KAPP
LR*	0.834	0.750	0.679	0.614	0.735	0.694	0.444
SVC*	0.844	0.767	0.699	0.605	0.726	0.703	0.454
XGB*	0.860	0.783	0.711	0.614	0.752	0.717	0.483
RF*	0.843	0.779	0.667	0.461	0.725	0.587	0.251
**SVC2**	0.835	0.759	0.679	0.603	0.722	0.695	0.452
**MAX**	0.853	0.783	0.699	0.613	0.731	0.711	0.476
**MIN**	0.850	0.771	0.689	0.603	0.738	0.692	0.444
**MEAN**	0.854	0.781	0.701	0.613	0.734	0.710	0.471
**MEDIAN**	0.849	0.778	0.691	0.593	0.721	0.699	0.448
*Mean*	0.847	0.772	0.691	0.591	0.732	0.690	0.436
*SD*	0.009	0.012	0.014	0.049	0.010	0.039	0.071
***StrokeClassifier***	***0.809***	***0.719***	***0.699***	***0.608***	***0.735***	***0.708***	***0.467***

(B) Class-wide performances by *StrokeClassifier*								
Training data	Testing data	Class	Accuracy	PPV	F1	Kappa	FPR	FNR
YNHH+MGH (N=1932)	MIMIC-RF (N=405)	1	0.844	**0.370**	0.860	**0.377**	**0.114**	0.042
		2	**0.780**	**0.841**	**0.782**	**0.535**	0.096	**0.123**
		3	**0.941**	0.385	**0.946**	0.424	**0.040**	**0.020**
		4	0.842	0.689	0.831	0.475	0.047	0.111
MGH (N=1002)	MIMIC-RF (N=405)	1	0.849	**0.369**	0.862	**0.357**	0.101	0.049
		2	**0.768**	**0.814**	**0.768**	**0.500**	**0.119**	**0.114**
		3	**0.956**	0.500	**0.956**	0.477	**0.022**	**0.022**
		4	0.844	0.688	0.835	0.490	0.049	0.106
	YNHH (N=930)	1	0.804	0.615	0.805	0.493	0.102	**0.094**
		2	**0.803**	**0.760**	**0.804**	**0.600**	**0.108**	0.089
		3	0.875	0.710	0.872	0.589	**0.051**	0.074
		4	**0.891**	**0.533**	**0.891**	**0.465**	0.053	**0.056**
YNHH (N=930)	MIMIC-RF (N=405)	1	0.820	**0.296**	0.837	**0.267**	**0.123**	0.057
		2	**0.765**	**0.845**	**0.768**	**0.511**	0.089	**0.146**
		3	**0.923**	0.314	**0.935**	0.379	**0.059**	**0.017**
		4	0.820	0.606	0.810	0.414	0.064	0.116
	MGH (N=1002)	1	**0.813**	0.718	**0.808**	0.527	0.069	**0.118**
		2	0.821	**0.767**	0.822	**0.637**	**0.105**	0.074
		3	0.878	**0.588**	0.882	0.564	0.075	**0.047**
		4	**0.888**	0.589	**0.886**	**0.502**	**0.051**	0.061

N.B. misclassification or error rate = 1 – accuracy; PPV = 1 – FDR.

**Table 7.** Application of *StrokeClassifier* to cryptogenic stroke patients

TOAST predicted	YNHH	MGH	MIMIC (RF-imputed)	Merged
1	55 (19.3%)	89 (21.8%)	26 (27.7%)	170 (21.6%)
2	93 (32.6%)	155 (37.9%)	24 (25.5%)	272 (34.5%)
3	30 (10.5%)	30 (7.3%)	5 (5.3%)	65 (8.2%)
4	19 (6.7%)	24 (5.9%)	13 (13.8%)	56 (7.1%)
Persistently cryptogenic	88 (30.9%)	111 (27.1%)	26 (27.7%)	225 (28.6%)
Total	285 (100%)	409 (100%)	94 (100%)	788 (100%)

## Data Availability

The electronic health record data of YNHH and MGH cannot be made available publicly. Sharing this data externally without proper consent could compromise patient privacy and would violate the Institutional Review Board approval for the study. MIMIC-III data is publicly available from the PhysioNet repository.

## ABBREVIATIONS:

AF: Atrial fibrillation
AIS: Acute ischemic stroke
AUC: Area under the curve
AUCROC: Area under the curve of the receiver operating characteristic
AUPRC: Area under the precision-recall curve
BA: Balanced accuracy
CUI: Concept Unique Identifier
EHR: Electronic health record
FDR: False discovery rate
FNR: False negative rate
FPR: False positive rate
HPO: Hyperparameter optimization
HRT: Heart-related features
HEX: Social history, NIHSS, vital signs features
KAPP: Cohen’s kappa
LAB: Laboratory test features
LR: Logistic regression
NIHSS: National Institutes of Health Stroke Scale
PR: Precision-Recall
PPV: Positive predictive value
PCA: Principal component analysis
RAD: Radiology-related features
ROC: Receiver operating characteristic
RF: Random Forests
SHAP: SHapley Additive explanation
SVC: Support vector classifier
TTE: Transthoracic echocardiography
UMLS: Unified Medical Language System
XGB: XGboost
