# Supplementary material for: StrokeClassifier: Ischemic Stroke Etiology Classification by Ensemble Consensus Modeling Using Electronic Health Records
Source: Res Sq. 2023 Oct 31:rs.3.rs-3367169. Preprint. [Version 1] doi: 10.21203/rs.3.rs-3367169/v1 (PMC10635373; doi:10.21203/rs.3.rs-3367169/v1)

Supplementary Figures

Figure S1. Exploratory data analyses of categorical and numerical features of 3 cohorts

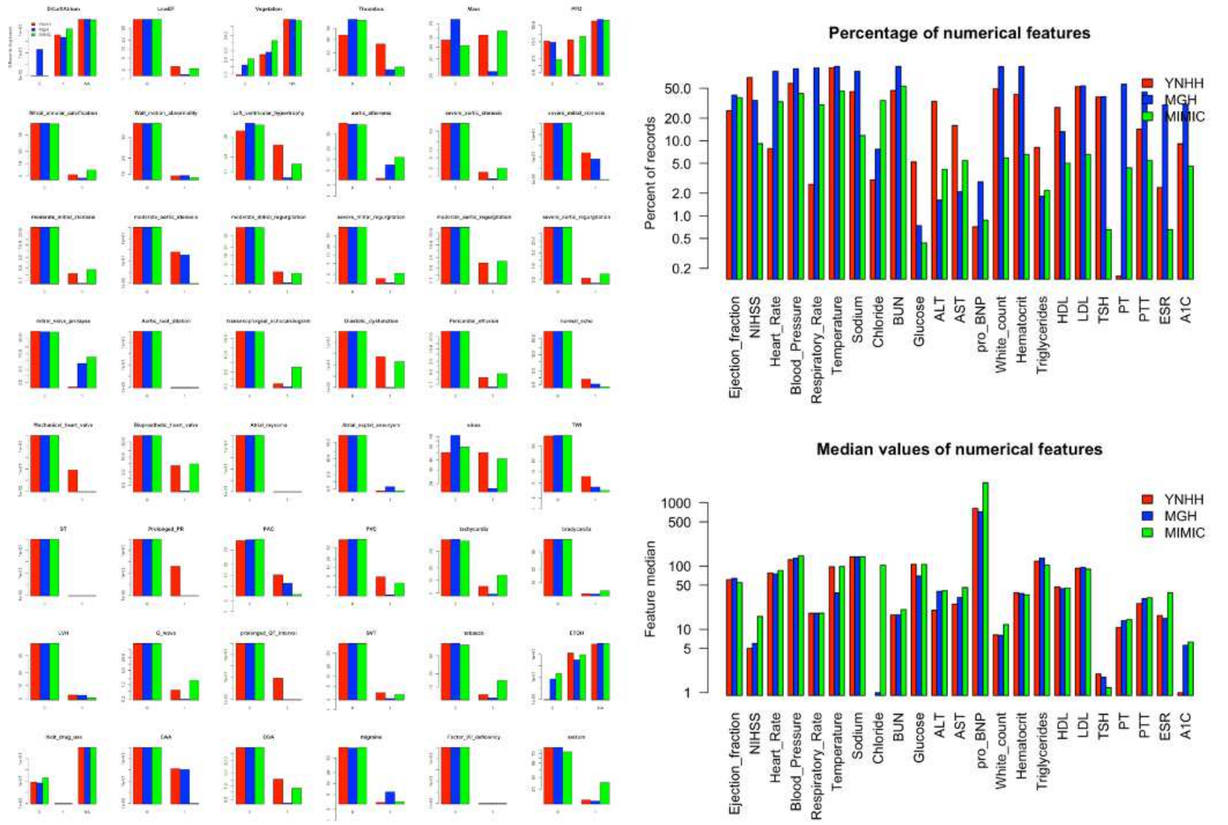

Figure S2. Performance comparisons among all feature groups and the 4 ML algorithms and between the training and validation sets of 5-fold cross validation. The bar plot is from LR*.

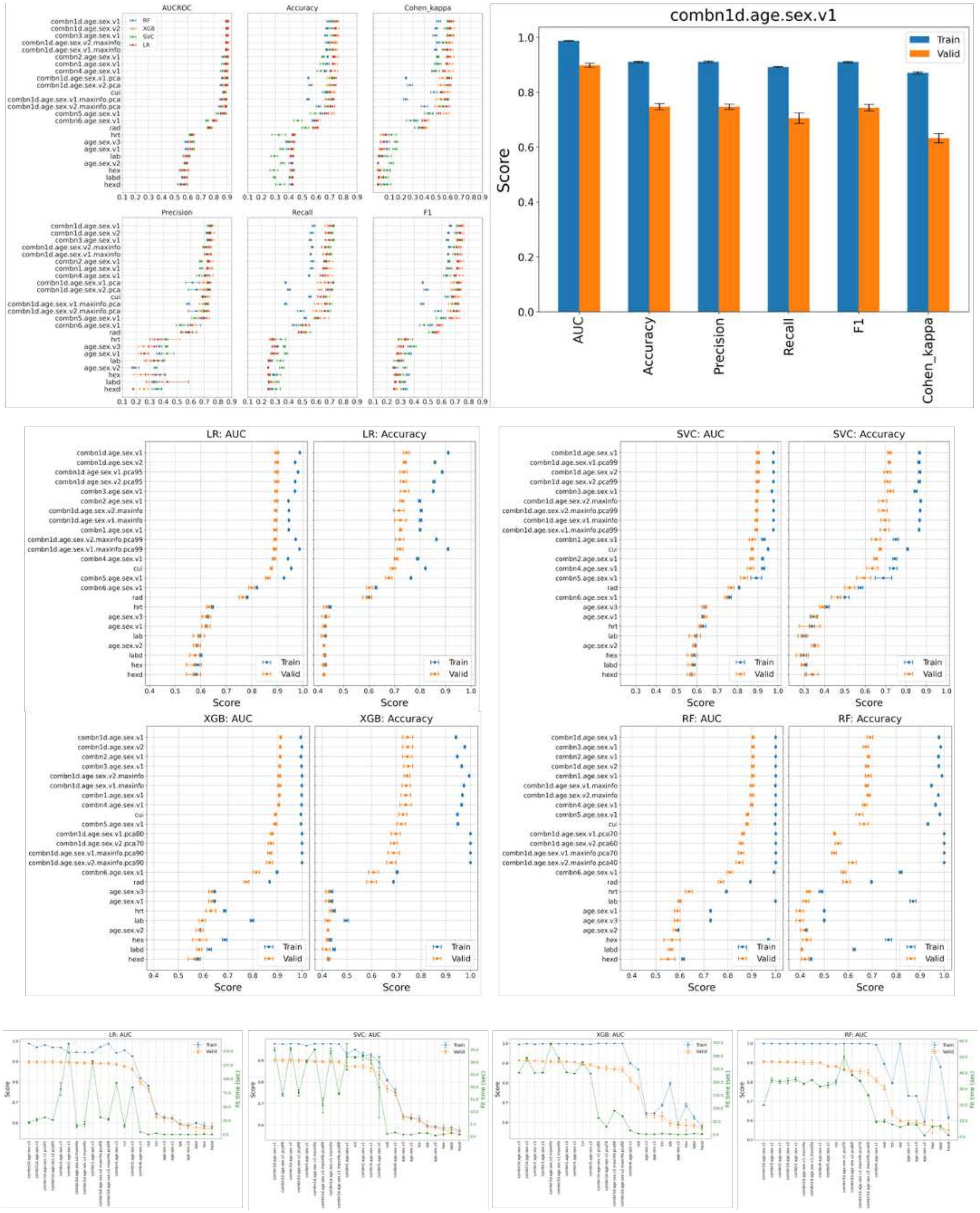

Figure S3. RMFCV300 performances in terms of AUCROC and AUPRC for each optimized model with combn1d.age.sex.v1

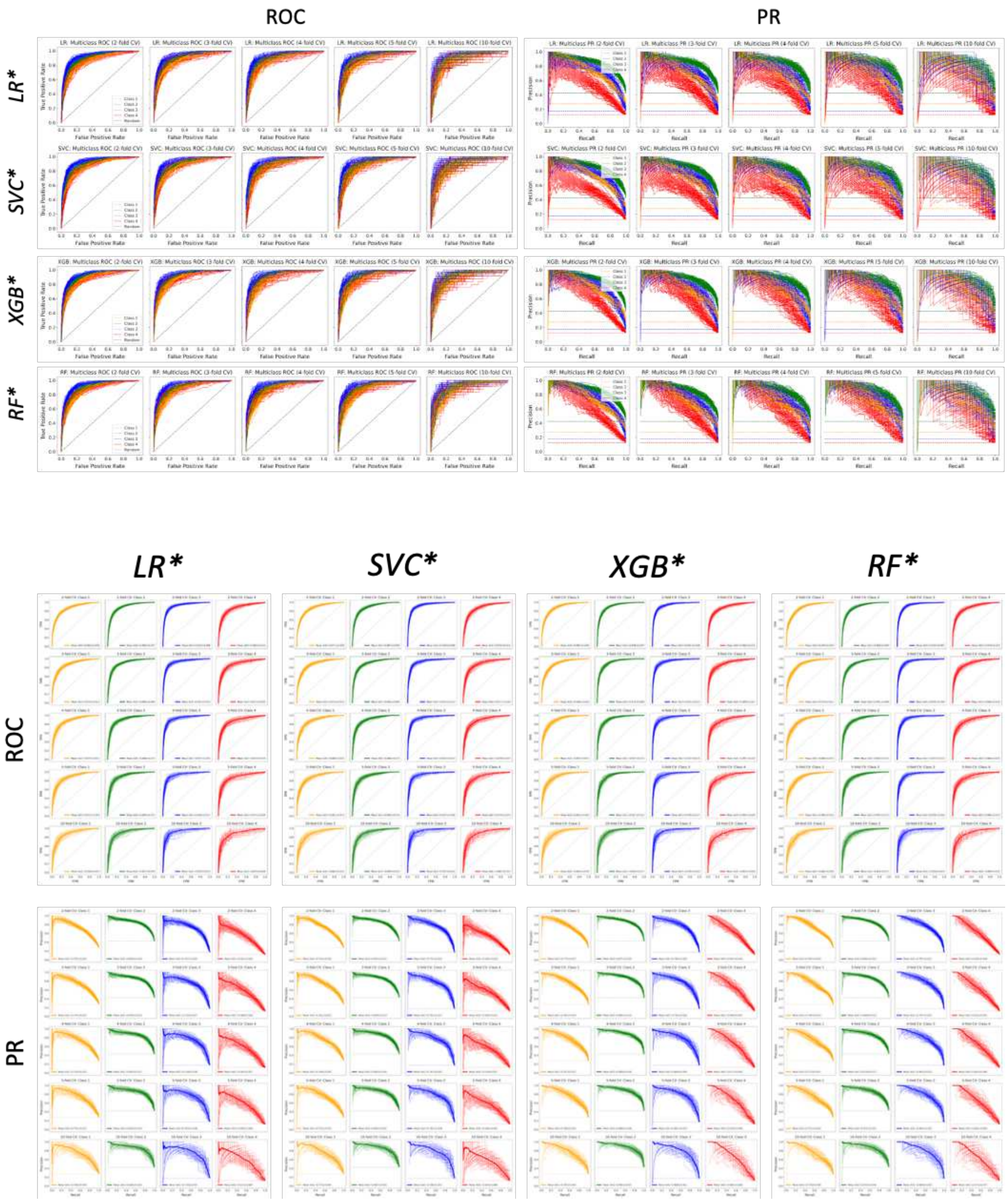

Figure S4. Frequency distributions of the top 10 features contributing to the top 5 PCs by SHAP analysis of the 4 PCA-based optimized models.

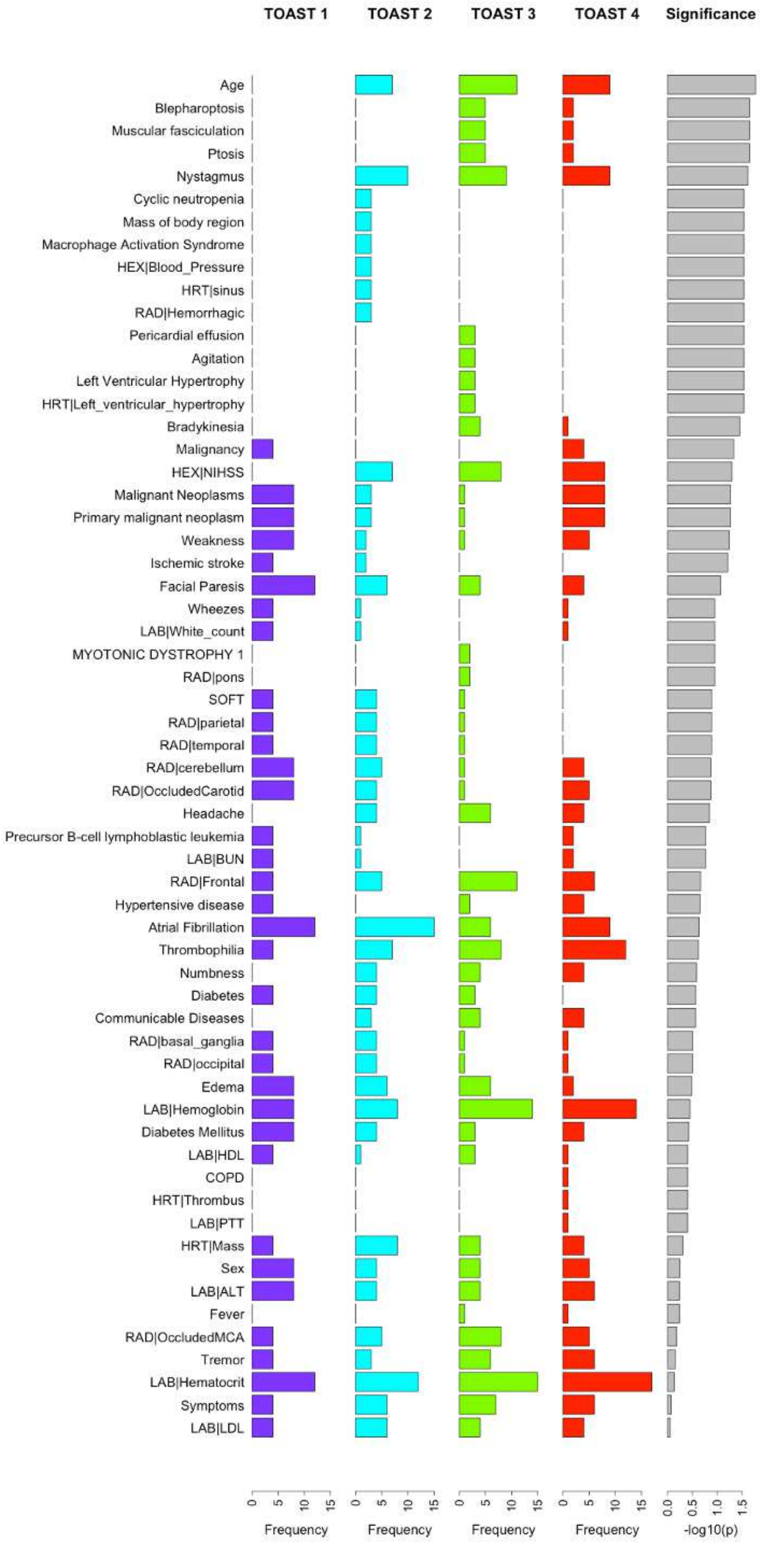

Figure S5. Correlation between SHAP analysis and statistical tests. (A) Feature correlations between mean(|SHAP|) averaged over the 4 optimized models for each class and the statistic, D (top row), and p-value (bottom row) by Kolmogorov-Smirnov tests for each class vs. the rest. The top 10 features are shown in dark red and Pearson correlation coefficients, *r*, on the top left. (B) Similar analyses to (A) by Student's t-tests.

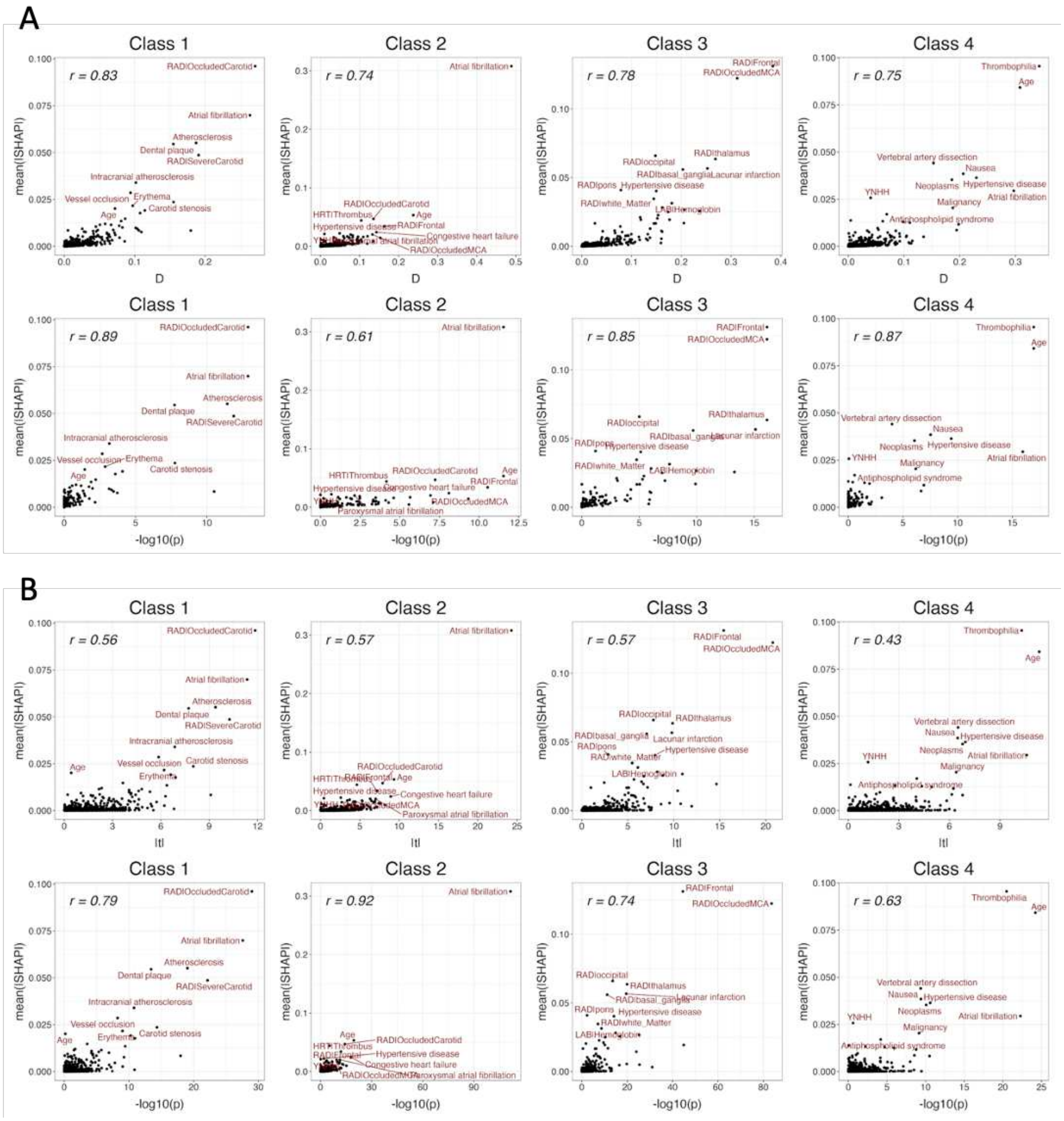

Supplement: Supplement 1 [file NIHPPrs3367169v1-supplement-1.pdf]
